# C/EBPα Activates Pre-existing and De Novo Macrophage Enhancers during Induced Pre-B Cell Transdifferentiation and Myelopoiesis

**DOI:** 10.1016/j.stemcr.2015.06.007

**Published:** 2015-07-30

**Authors:** Chris van Oevelen, Samuel Collombet, Guillermo Vicent, Maarten Hoogenkamp, Cyrille Lepoivre, Aimee Badeaux, Lars Bussmann, Jose Luis Sardina, Denis Thieffry, Miguel Beato, Yang Shi, Constanze Bonifer, Thomas Graf

**Affiliations:** 1 Center for Genomic Regulation and Pompeu Fabra University, 08003 Barcelona, Spain; 2 Ecole Normale Supérieure, Institut de Biologie de l’ENS, INSERM, U1024, Centre National de la Recherche Scientifique (CNRS) 8197, 75005 Paris, France; 3 School of Cancer Sciences, Institute of Biomedical Research, University of Birmingham, Birmingham B15 2TT, UK; 4 CNRS, Aix-Marseille Université, IGS UMR7256, 13288 Marseille, France; 5 Harvard Medical School, Children’s Hospital, Boston, MA 02115, USA

## Abstract

Transcription-factor-induced somatic cell conversions are highly relevant for both basic and clinical research yet their mechanism is not fully understood and it is unclear whether they reflect normal differentiation processes. Here we show that during pre-B-cell-to-macrophage transdifferentiation, C/EBPα binds to two types of myeloid enhancers in B cells: pre-existing enhancers that are bound by PU.1, providing a platform for incoming C/EBPα; and de novo enhancers that are targeted by C/EBPα, acting as a pioneer factor for subsequent binding by PU.1. The order of factor binding dictates the upregulation kinetics of nearby genes. Pre-existing enhancers are broadly active throughout the hematopoietic lineage tree, including B cells. In contrast, de novo enhancers are silent in most cell types except in myeloid cells where they become activated by C/EBP factors. Our data suggest that C/EBPα recapitulates physiological developmental processes by short-circuiting two macrophage enhancer pathways in pre-B cells.

## Highlights

• C/EBPα activates two classes of prospective myeloid enhancers in B cells
• Pre-existing enhancers are bound by PU.1 and become hyper-activated by C/EBPα
• C/EBPα acts as a pioneer factor with delayed kinetics on de novo enhancers
• The two types of enhancers direct myeloid cell fate in B cells and hematopoiesis

## Graphical Abstract

**Figure:**
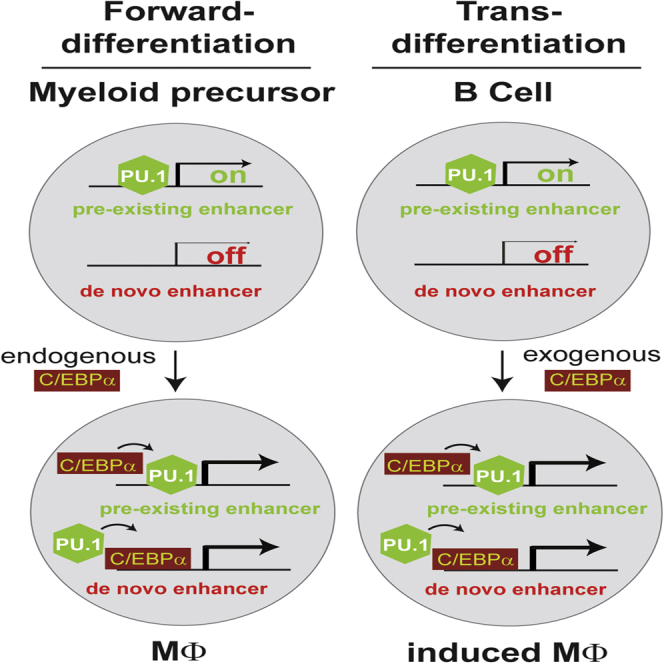

## Teaser

In this study, Graf and colleagues show that, during B-cell-to-macrophage transdifferentiation, C/EBPα activates two types of myeloid enhancers in B cells. The two enhancer types are differentially activated, dictating gene expression levels of macrophage genes during the conversion of B cells into macrophages and normal hematopoiesis.

## Introduction

The discovery that transcription factors (TFs) can convert somatic cells into both specialized and induced pluripotent stem cells (iPSCs) has revolutionized stem cell research and promises to have major clinical applications (Graf and Enver, 2009; Yamanaka and Blau, 2010). Lineage-instructive TFs activate and repress tissue-specific genes by recognizing sequence-specific DNA consensus motifs contained within enhancers and promoters (Ptashne, 2007). They establish gene regulatory networks (GRNs) of the novel gene expression program while dismantling those of the old program, involving the formation of feedforward, cross-inhibitory, and auto-regulatory loops (Bertrand and Hobert, 2010; Davidson, 2010; Graf and Enver, 2009; Holmberg and Perlmann, 2012). However, how these processes are coordinated and whether they recapitulate normal development remain unclear (Vierbuchen and Wernig, 2011), especially as neither TF-induced lineage conversions nor iPSC reprogramming appear to retrace normal developmental pathways (Apostolou and Hochedlinger, 2013; Di Tullio et al., 2011; Ladewig et al., 2013; Vierbuchen and Wernig, 2011).

Lineage-instructive TFs act through synergistic and cross-antagonistic interactions, are typically able to access closed chromatin (Zaret and Carroll, 2011), preferentially target sites with specific histone mark combinations, and bind to either nucleosome-depleted or nucleosome-dense regions (Soufi et al., 2012; Taberlay et al., 2011; Wapinski et al., 2013). However, what establishes these chromatin configurations in the first place and what proportion of the incoming reprogramming factors interacts with pre-existing TF complexes are largely unknown. A major reason for these gaps in our knowledge is that cell conversion frequencies in most cell systems are low, complicating efforts to study early events in a time-resolved fashion.

An exception is the transdifferentiation of pre-B/B cells into macrophages induced by the leucine zipper-type TF C/EBPα, which is arguably the most efficient and rapid system described so far (Bussmann et al., 2009; Di Tullio and Graf, 2012; Xie et al., 2004). C/EBPβ, like C/EBPα, can likewise induce B cell transdifferentiation into macrophages (Bussmann et al., 2009; Xie et al., 2004), but the two factors also have non-redundant functions. Mice ablated for C/EBPα die shortly after birth because they lack granulocyte/macrophage progenitors (GMPs, precursors of neutrophil granulocytes and macrophages, two closely related myeloid cell types) as well as granulocytes, while C/EBPβ-knockout animals are fully viable but contain macrophages and B cells with functional defects (Chen et al., 1997; Tanaka et al., 1995). C/EBPα cooperates with PU.1 (Spi1) to regulate myeloid gene expression (Friedman, 2007), the two factors interact physically (Reddy et al., 2002), and a combination of C/EBPα and PU.1 converts fibroblast into macrophage-like cells (Feng et al., 2008). The *Pu.1* gene encodes an Ets family TF specifically expressed in the early stages of hematopoiesis and its knockout generates mice that lack both myeloid and lymphoid cells (Scott et al., 1994). Low-level expression of PU.1 in hematopoietic precursors induces B cell differentiation, whereas high levels favor myeloid differentiation (DeKoter and Singh, 2000).

Here we have analyzed, in a time-resolved manner, how C/EBPα establishes a myeloid expression program in pre-B cells, and we found that it binds to both pre-existing enhancers occupied by PU.1 and de novo enhancers where it acts as a pioneer factor. Strikingly, the combined activation of these enhancer types, regulating the expression of nearby macrophage genes, recapitulates the activation of myeloid enhancers and associated genes during normal hematopoiesis.

## Results

### C/EBPα Induces High-Level Expression of *Pu.1* and *Cebpb*

**Figure 1:**
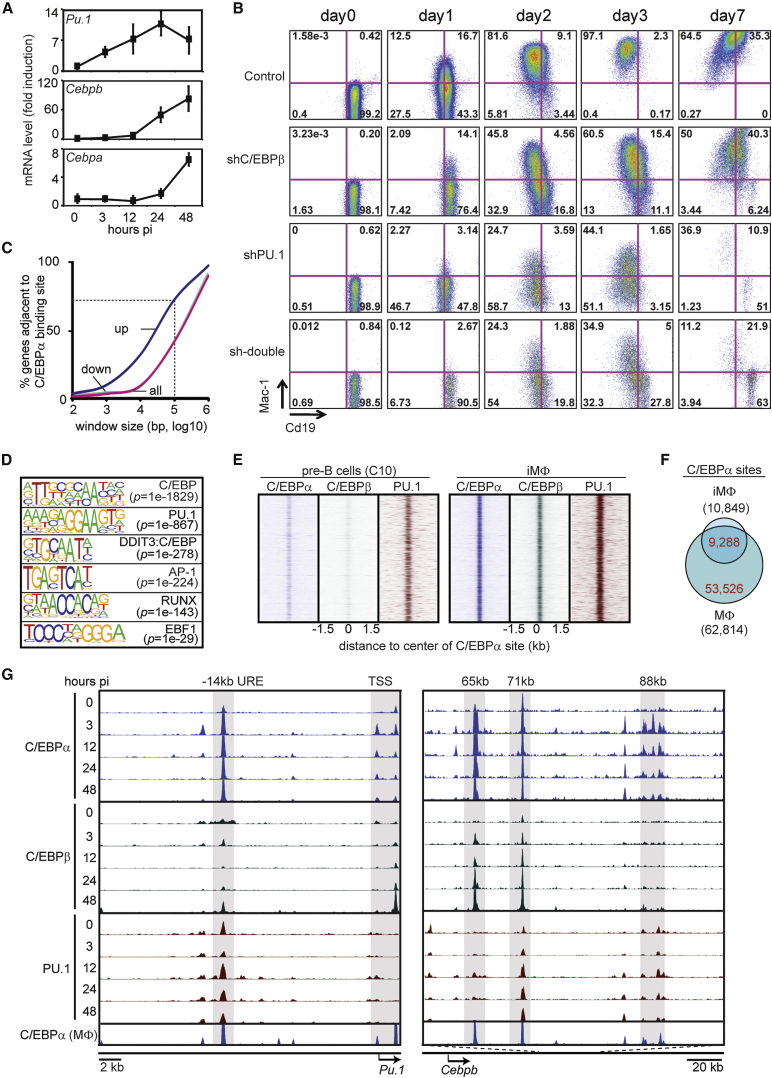
Upregulation of *Cebpb* and *Pu.1* Genes by C/EBPα and Effects of Their Knockdown on Transdifferentiation (A) Expression of endogenous *Pu.1*, *Cebpb*, and *Cebpa* RNA after β-Est induction of C10 cells as measured by qRT-PCR. Data are represented as mean ± SEM (independent triplicates) expressed as the fold induction relative to uninduced pre-B cells. (B) FACS plots of C11 pre-B cell carrying either a scrambled short hairpin knockdown construct (control) or constructs against C/EBPβ, PU.1, or both, and induced by β-est treatment. See also Figure S1C. (C) Percentage of upregulated or downregulated genes (>2-fold) within defined windows around C/EBPα sites. Dotted lines indicate that 70% of all upregulated genes are within 100 kb of a C/EBPα-binding site. (D) Significantly enriched sequence motifs at C/EBPα-binding sites as determined by HOMER. (E) Heatmaps visualizing C/EBPα, C/EBPβ, and PU.1 binding in pre-B cells and iMΦ. Window, 3 kb; bin, 10 bp. See also Figure S1E. (F) Venn diagram showing the intersection of C/EBPα sites in iMΦ (n = 10,849) and primary MΦ (n = 62,814). (G) Screenshots of C/EBPα, C/EBPβ, and PU.1 binding at selected enhancers in C10 cells and of C/EBPα in primary MΦ. Arrows indicate TSS, length of ORF, and direction of transcription. See also Figure S1I.

To study how C/EBPα induces transdifferentiation, we used two pre-B cell lines that express an inducible C/EBPαER fusion protein tagged with either human CD4 (hCD4; C11 cells) or GFP (C10 cells). In both lines, treatment with 17 beta-estradiol (β-Est) shuttles C/EBPαER into the nucleus and induces the formation of macrophage-like cells within 2 to 3 days (Bussmann et al., 2009). Importantly, C/EBPα mRNA levels in C10 cells at 0 hr post-induction (hpi) or 24 hpi did not exceed C/EBPα levels observed in primary macrophages (MΦ) (Figure S1A). To monitor two important myeloid regulators known to cooperate with C/EBPα, we tested the expression levels of *Cebpb* and *Pu.1*. These genes were expressed at low to intermediate levels in pre-B cells (Figure S1B) and became upregulated within 3–12 hpi (Figure 1A). As C10 cells become transgene independent 24 hpi (Bussmann et al., 2009), i.e., before the expression of endogenous C/EBPα (Figure 1A), we determined whether the rapid activation of C/EBPβ and PU.1 is necessary for transdifferentiation. We generated C11 cells stably expressing a short hairpin RNA (shRNA) against C/EBPβ, PU.1, or both. Cells were induced with β-Est and analyzed by fluorescence-activated cell sorting (FACS) for the presence of Cd19 and Mac-1 (CD11b) at different days thereafter. At 3 days post-induction (dpi), the knockdown of C/EBPβ and of PU.1 resulted in a 35% and 50% reduction in the formation of Mac-1^+^Cd19^−^ cells, respectively, while deleting both factors further enhanced the effect. At 7 dpi, Mac-1 expression in shC/EBPβ cells caught up with wild-type levels, whereas cells expressing shPU.1 exhibited extensive cell death (Figures 1B and S1C). These data show that C/EBPα rapidly upregulates *Pu.1* and *Cebpb,* that PU.1 is necessary to establish the myeloid GRN, and that C/EBPβ plays a more minor role.

### A Limited Set of Sites Stably Bound by C/EBPα Correlates with the Upregulation of Macrophage Genes

To explore the mechanism by which C/EBPα turns on the myeloid program in pre-B cells, we treated C10 cells for different times with β-Est and performed chromatin immunoprecipitation followed by deep sequencing (ChIP-seq) experiments, using antibodies to C/EBPα, C/EBPβ, and PU.1 (Table S1 gives a summary of ChIP-seq results and peak calling). A total of 54,198 non-redundant C/EBPα-enriched regions could be detected during the time course of which 10,849 sites were stably bound (i.e., up to 48 hpi, Table S2), whereas the remaining sites were transiently bound. Genes nearest stable binding sites, but not transient sites, were enriched for upregulated genes (Figure S1D). In addition, using a sliding-window approach, we observed that 70% of upregulated genes were localized within 100 kb of a stable C/EBPα-binding site, whereas no such enrichment was seen for downregulated genes (Figure 1C). Motif analysis of the stable sites in 48-hpi cells (hereafter referred to as induced macrophages or iMΦ) showed strong enrichment for consensus motifs of C/EBP and PU.1. The same sites also were enriched for AP-1 (Jun and Fos) and RUNX motifs, as previously reported (Figure 1D; Heinz et al., 2010) and more weakly enriched for EBF1 (Figure 1D; also see Figure 3). The majority of stable C/EBPα sites were co-occupied by C/EBPβ and PU.1 in iMΦ, and ∼40% of these were pre-bound by PU.1 in pre-B cells, however, showing lower intensity signals (Figure 1E). Low-intensity signals in pre-B cells also were detectable for C/EBPβ, reflecting its low-level expression, as well as for C/EBPα (Figure S1E), suggesting some leakiness of the transgene.

A total of 10,849 C/EBPα sites were detected in iMΦ and 62,814 in bone-marrow-derived macrophages (MΦ) (Zhang et al., 2013), showing 9,288 common sites (Figure 1F). The larger number of sites in MΦ compared to iMΦ cannot be explained by a higher sequencing depth (Table S1). However, these differences became smaller when the numbers of associated genes were compared as follows: C/EBPα sites combined with 5,849 and 14,078 genes in iMΦ and MΦ, respectively, and shared 5,252 genes (Figure S1F). The shared gene set was enriched for genes that became upregulated during transdifferentiation of primary B cells into macrophages (Di Tullio et al., 2011), whereas the gene set unique for MΦ (8,826) was actually depleted (Figure S1G). In addition, shared upregulated genes were enriched for gene ontology (GO) terms associated with myeloid function, while upregulated genes unique for MΦ were not (Figure S1H). The induced rapid and efficient conversion of pre-B cells into highly motile, aggregating, and phagocytic macrophages within 51 hr (Movie S1; Bussmann et al., 2009) further supports the interpretation that C/EBPα binds to a core set of enhancers in iMΦ required for myeloid cell specification.

The binding of C/EBPα, C/EBPβ, and PU.1 in induced C10 cells and of C/EBPα in primary MΦ is illustrated for the promoter and the −14-kb URE enhancer of the *Pu.1* gene (Yeamans et al., 2007), for three putative enhancers of *Cebpb* (Figure 1G), as well as for putative enhancers of *Fos* and *Il1b* (Figure S1I). (Genomic coordinates of these and other regions are described in Table S3.) Together, our data suggest that C/EBPα combined with C/EBPβ and PU.1 activates a core set of enhancers, shared between the cell line and primary macrophages, required to induce macrophage specification.

### Prospective Myeloid Enhancers in Pre-B Cells Fall into Two Broad Classes

**Figure 2:**
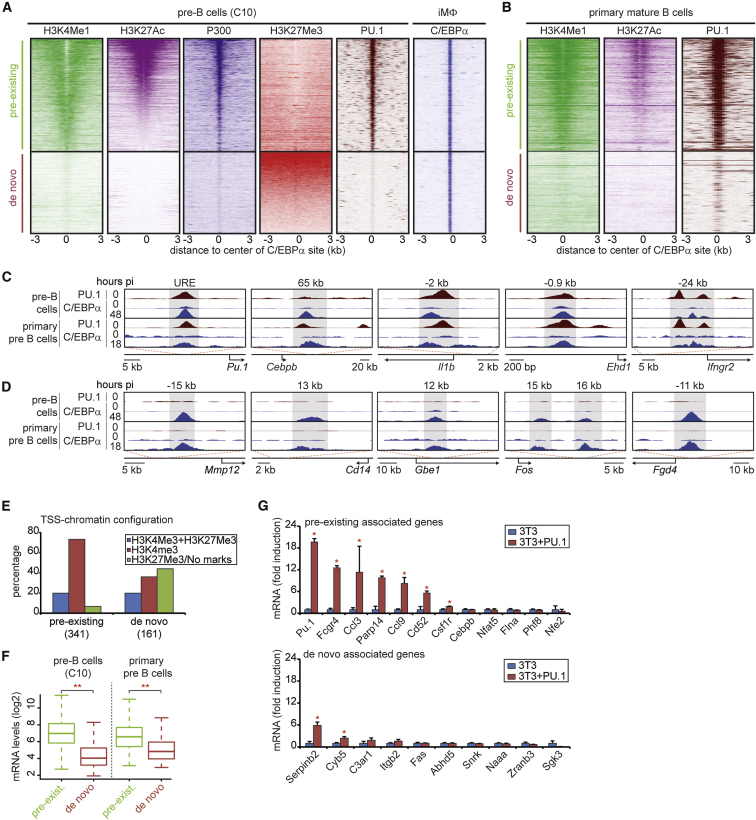
Prospective Myeloid Enhancers with Either Pre-existing or De Novo Configurations in Pre-B Cells (A) Heatmaps visualizing H3K4Me1, H3K27Ac, P300, H3K27Me3, and PU.1 in pre-B cells and C/EBPα binding in iMΦ. Center of C/EBPα binding, 0; window, 6 kb; bin, 100. See also Figures S2B–S2D. (B) As in (A), visualizing H3K4Me1, H3K27Ac, and PU.1 in primary mature B cells. (C and D) Screenshots of C/EBPα and PU.1 binding in C10 cells (0 and 48 hpi) and primary pre-B cells (0 and 18 hpi) at pre-existing (C) and de novo enhancers (D). (E) Distribution of genes nearest pre-existing or de novo enhancers marked with bivalent (H3K4Me3, H3K27Me3), active (H3K4Me3), or repressed (H3K27Me3 or no marks) promoters. See also Figure S2E. (F) Distribution of mRNA levels of upregulated genes nearest to either pre-existing (n = 318) or de novo (n = 103) enhancers in pre-B cells (C10) and primary pre-B cells. Statistical analysis by Wilcoxon rank-sum test, ^∗∗^p < 0.001. (G) Expression of genes nearest pre-existing or de novo enhancers in 3T3 cells or 3T3 overexpressing PU.1 by qRT-PCR. Data are represented as mean ± SEM (independent triplicates) and expressed as the fold induction relative to 3T3 cells. Statistical analysis by Student’s t test, ^∗^p < 0.05.

To characterize the epigenetic status of prospective myeloid regulatory regions in pre-B cells, we performed ChIP-seq experiments for histone modifications characteristic of poised (H3K4Me1), active (H3K27Ac, P300), and repressed enhancers (H3K27Me3) (Creyghton et al., 2010; Rada-Iglesias et al., 2011), and we analyzed levels of these marks at C/EBPα sites away from the transcription start site (TSS), representing putative enhancers (Figure S2A). We observed two broad classes of prospective myeloid enhancers in pre-B cells as follows: (1) pre-existing enhancers that were decorated with H3K4Me1, H3K27Ac, and P300 and depleted for H3K27Me3 (Figure 2A); and (2) de novo enhancers that lacked any of the active enhancer marks but were instead often decorated with H3K27Me3 (Figure 2A). Similar results were obtained by performing ChromHMM analysis (Figure S2C; Ernst and Kellis, 2012) as an independent analytical approach demonstrating that pre-existing enhancers are enriched for activation marks, whereas de novo enhancers are depleted for activation marks and enriched for H3K27Me3 (Figure S2D). Approximately two-thirds of the pre-existing enhancers were bound by PU.1 and exhibited high levels of activation marks compared to sites not bound by PU.1 (Figure S2B). Importantly, we confirmed the presence of pre-existing- and de-novo-type enhancers in primary B cells by using recently published ChIP-seq datasets (Figure 2B; Heinz et al., 2010; Lara-Astiaso et al., 2014). Furthermore, we confirmed binding of C/EBPα to selected pre-existing enhancers of the *Pu.1*, *Cebpb*, *Il1b*, *Ehd1*, and *Ifngr2* genes and to de novo enhancers of the *Mmp12*, *Cd14*, *Gbe1*, *Fos*, and *Fgd4* genes in primary pre-B cells induced to transdifferentiate (Figures 2C and 2D; Di Stefano et al., 2014).

To determine whether the enhancer activation state in pre-B cells correlates with gene expression, we first analyzed the promoter configuration of adjacent genes and found the following: 73% of the pre-existing enhancers paired with active promoters (as defined by the sole presence of H3K4Me3), 7% were decorated with inactive promoters (H3K27Me3 or no marks), and 20% were decorated with promoters containing a bivalent domain (H3K4Me3 and H3K27Me3) (Figure 2E). In contrast, only 36% of de novo enhancers paired with active promoters, 44% with inactive promoters, and 20% with bivalent promoters (Figures 2E and S2E). Based on these results, we re-defined pre-existing enhancers as those that combine with active promoters and de novo enhancers as those that pair with inactive promoters. As expected, genes associated with pre-existing enhancers already were expressed at significant levels in pre-B cells and primary pre-B cells (Bussmann et al., 2009; Di Tullio et al., 2011), whereas genes associated with de novo enhancers only showed background expression levels (Figure 2F).

The finding that pre-B cells express genes associated with pre-existing enhancers predicts that PU.1 expression in cells devoid of PU.1 will selectively activate these genes. To test this, we expressed PU.1 in 3T3 fibroblasts (Figure 2G) and measured mRNA levels of a number of genes associated with either pre-existing or de novo enhancers. Supporting the hypothesis that PU.1 preferentially binds to pre-existing enhancers and activates associated genes, we observed that 6 of 11 pre-existing enhancer-associated genes tested were upregulated as compared to 2 of 10 de novo genes (Figure 2G; primer sequences are in Table S4).

Together, our findings suggest that C/EBPα is capable of activating two broad classes of prospective myeloid enhancers in pre-B cells as follows: (1) pre-existing enhancers with active enhancer marks that are predominantly associated with expressed genes, and (2) de novo enhancers lacking such marks that are predominantly associated with silenced genes.

### A Subset of Pre-existing Myeloid Enhancers Is Bound by the B Cell TF Ebf1 in Pre-B Cells

**Figure 3:**
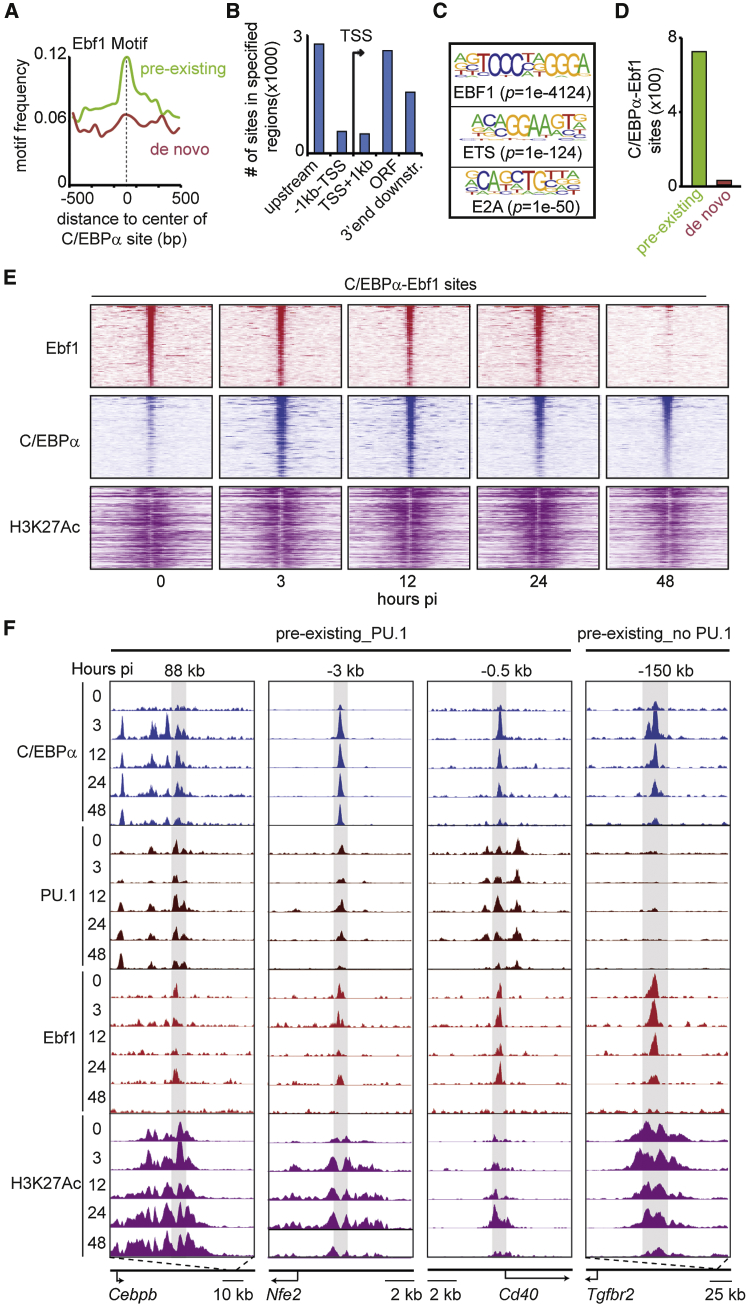
Binding of the B Cell TF Ebf1 to Pre-existing Enhancers (A) Frequency of Ebf1 motif within pre-existing and de novo C/EBPα-binding sites by HOMER. (B) Genomic distribution of Ebf1-binding events (n = 6,627) relative to the TSS in C10 cells. ORF, open reading frame. (C) Significantly enriched sequence motifs at Ebf1-binding sites, as determined by HOMER. (D) Number of C/EBPα sites bound by Ebf1 for each enhancer type. (E) Heatmaps, centered on C/EBPα binding in iMΦ, visualizing Ebf1, C/EBPα, and H3K27Ac after the induction of transdifferentiation. Center of binding, 0; window, 6 kb; bin, 100. (F) Screenshots of C/EBPα, PU.1, Ebf1, and H3K27Ac ChIP-seq profiles at selected enhancer regions in C10 cells. See also Figures S3A–S3D.

Our finding that motifs associated with the B cell TF Ebf1 are enriched in myeloid enhancers (see Figure 1D) prompted us to study their relevance in transdifferentiation. Analysis of the Ebf1 motif distribution shows that it is specifically enriched in pre-existing enhancers (Figure 3A). To test actual binding of Ebf1, we performed ChIP-seq experiments in pre-B cells yielding 6,627 Ebf1 peaks that were predominantly located in intergenic regions (Figure 3B) and enriched for EBF1, ETS (PU.1), and E2A motifs (Figure 3C). In line with the motif analysis, intersection of Ebf1-bound sites with the two types of myeloid enhancers showed 725 that were associated with pre-existing enhancers, but virtually none with de novo enhancers (Figure 3D).

To determine the functional state of the Ebf1-bound enhancers targeted by C/EBPα, we determined the kinetics of Ebf1 and C/EBPα binding as well as H3K27Ac decoration after induction of transdifferentiation. The heatmaps in Figure 3E show that, while C/EBPα binding already was observed after 3 hpi, the loss of Ebf1 binding was not detected until 48 hpi. However, H3K27Ac enrichment at these enhancers was maintained throughout the time course (Figure 3E), suggesting that the relevant enhancers remain active even after the loss of Ebf1. Examples of enhancers bound by Ebf1, C/EBPα, and PU.1 are shown in Figure 3F. This includes the 88-kb putative enhancer of *Cebpb* (see Figure 1G), as well as *Nfe2* and *Cd40* enhancers. In addition to enhancers bound by Ebf1, C/EBPα, and PU.1, 27% lack PU.1 binding, as exemplified by the −150-kb *Tgfbr2* putative enhancer (Figure 3F; additional examples are shown in Figure S3A). Ebf1 binding to these regions was confirmed using an independent Ebf1 ChIP-seq dataset (Treiber et al., 2010; Figures S3B and S3C). Importantly, genes associated with putative Ebf1-C/EBPα-bound enhancers were upregulated during transdifferentiation (Figure S3D).

In conclusion, a significant proportion of pre-existing myeloid enhancers targeted by C/EBPα in pre-B cells are bound by the B cell TF Ebf1. This finding raises the possibility that pre-existing myeloid enhancers act as bona fide B cell enhancers and that C/EBPα converts them into enhancers active in myeloid cells.

### C/EBPα Acts Both as a Pioneer and as a Secondary Factor at Prospective Myeloid Enhancers

**Figure 4:**
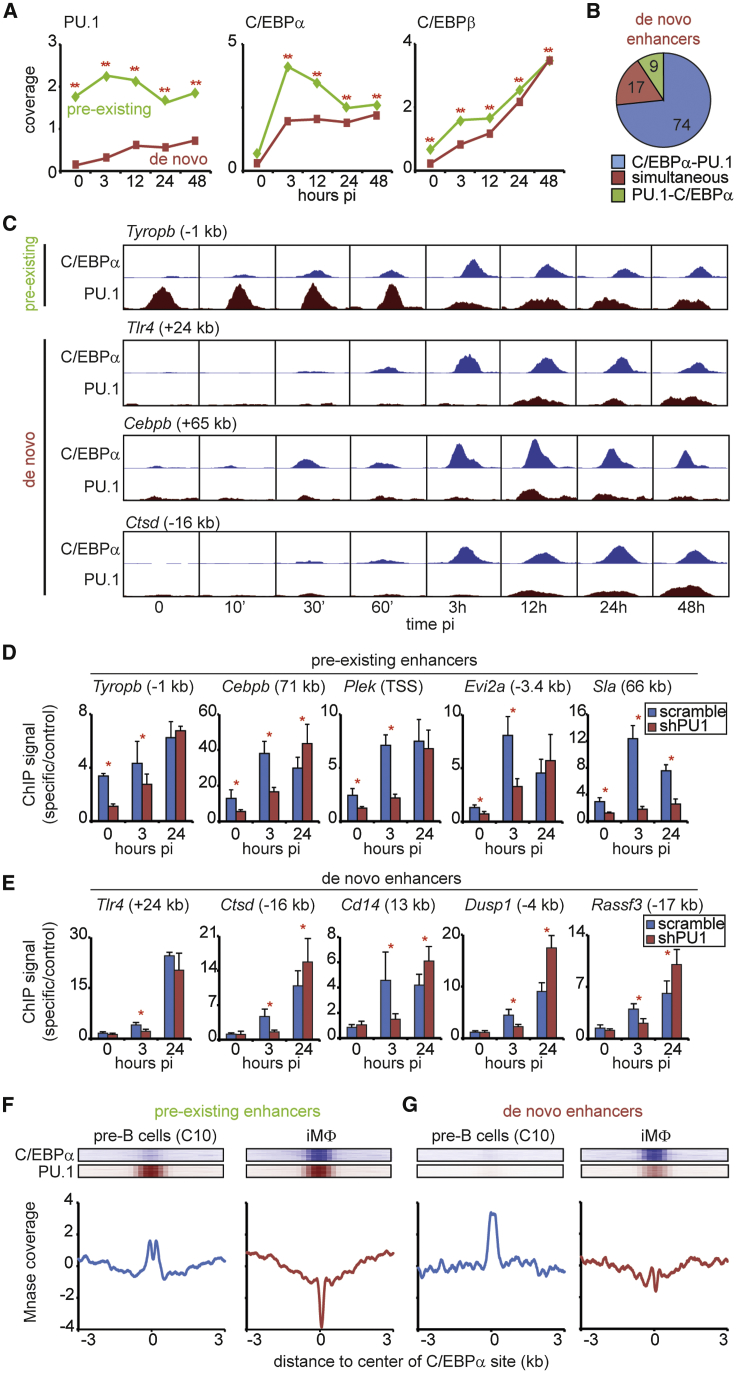
C/EBPα Binds to Open and Closed Chromatin (A) Kinetics of PU.1, C/EBPα, and C/EBPβ binding at center position of pre-existing (n = 4,711) and de novo (n = 3,424) enhancers at indicated hpi. Statistical analysis by Wilcoxon rank-sum test, ^∗∗^p < 0.001. See also Figures S4A and S4B. (B) C/EBPα and PU.1 binding order on de novo sites (C/EBPα first [C/EBPα-PU.1], simultaneously, or PU.1 first [PU.1- C/EBPα]. (C) Screenshots of C/EBPα and PU.1 binding at selected pre-existing or de novo enhancers (10, 30, and 60 min). (D and E) C/EBPα binding at pre-existing (D) or de novo (E) enhancers in induced C10 cells knocked down for PU.1. See also Figure S4C. Data are represented as mean ± SEM (independent triplicates). Statistical analysis by Student’s t test, ^∗^p < 0.05. Primer sequences are given in Table S4. (F and G) Average MNase profiles at pre-existing (F) and de novo (G) enhancers bound by C/EBPα and PU.1 (shown in blue and brown). Profiles were centered on PU.1 binding in iMΦ and normalized by median subtraction. Window, 6 kb; bin, 1 bp. See also Figures S4D and S4E.

To study how the two enhancer types become activated, we determined the binding kinetics of PU.1, C/EBPα, and C/EBPβ. As expected, at pre-existing enhancers PU.1 was bound throughout the time course, whereas it was initially absent at de novo enhancers, gradually increasing after induction (Figures 4A and S4A). In contrast, C/EBPα binding showed a steeper increase at pre-existing than at de novo enhancers, with both converging at 48 hpi and the rate of C/EBPα binding kinetics correlating with the starting levels of H3K27Ac or H3K27Me3, respectively (Figure S4B). Finally, C/EBPβ occupancy increased steadily at the two enhancer types (Figures 4A and S4A).

The binding profiles observed predict that at de novo enhancers C/EBPα binds before PU.1. Indeed, ChIP-seq experiments with induced C10 cells at early time points (10, 30, and 60 min post-induction) showed that C/EBPα binds to 74% of de novo sites *before* PU.1 (Figure 4B). An example of a putative pre-existing enhancer bound by PU.1 first is shown for the *Tyrobp* gene; examples of de novo enhancers are the 24-kb site of *Tlr4*, the 65-kb enhancer of *Cebpb*, and the −16-kb site of *Ctsd* (Figure 4C).

To further study the interplay between PU.1 and C/EBPα, we knocked down PU.1 in pre-B cells (Figure S4C), induced transdifferentiation for 3 and 24 hr, and analyzed C/EBPα binding at five pre-existing and five de novo enhancers. In control cells we observed higher binding of C/EBPα at 3 hpi for the pre-existing relative to the de novo enhancers. In addition, knockdown of PU.1 caused an initial decrease of C/EBPα binding at 3 hpi for both enhancer types on all loci tested (Figures 4D and 4E; primer sequences are in Table S4). However, at 24 hpi, C/EBPα binding recovered to control levels or even above in 9 of 10 enhancers tested (Figures 4D and 4E). This suggests that C/EBPα binding at pre-existing enhancers does not strictly require PU.1, raising the possibility that C/EBPα can access closed chromatin (see also Figures 2A and S4B).

To test this more directly, we performed micrococcal nuclease (Mnase) digestion experiments with chromatin isolated from pre-B cells and iMΦ cells and deep-sequenced nuclease-protected DNA. Average nucleosome profiles calibrated with sites uniquely bound by PU.1 revealed a nucleosome-depleted region (valley) flanked by two positioned nucleosomes (Figure S4D) that confirmed an earlier report (Heinz et al., 2010). Pre-existing enhancers bound by PU.1 in pre-B cells showed a small valley that became more pronounced in iMΦ (Figure 4F). In contrast, de novo enhancers targeted by C/EBPα in pre-B cells were contained in a nucleosome-dense region that changed into a profile similar to that observed for pre-existing enhancers, although less pronounced, in iMΦ (Figure 4G). No ordered nucleosome patterns were obtained with profiles centered on random genomic positions (Figure S4E).

Our data show that C/EBPα binds to a nucleosome-depleted region in pre-existing enhancers and to a nucleosome-dense region in de novo enhancers. These findings support the notion that C/EBPα can act as a pioneer factor.

### The C/EBPα and PU.1 Binding Order Determines the Activation Kinetics of Adjacent Genes

**Figure 5:**
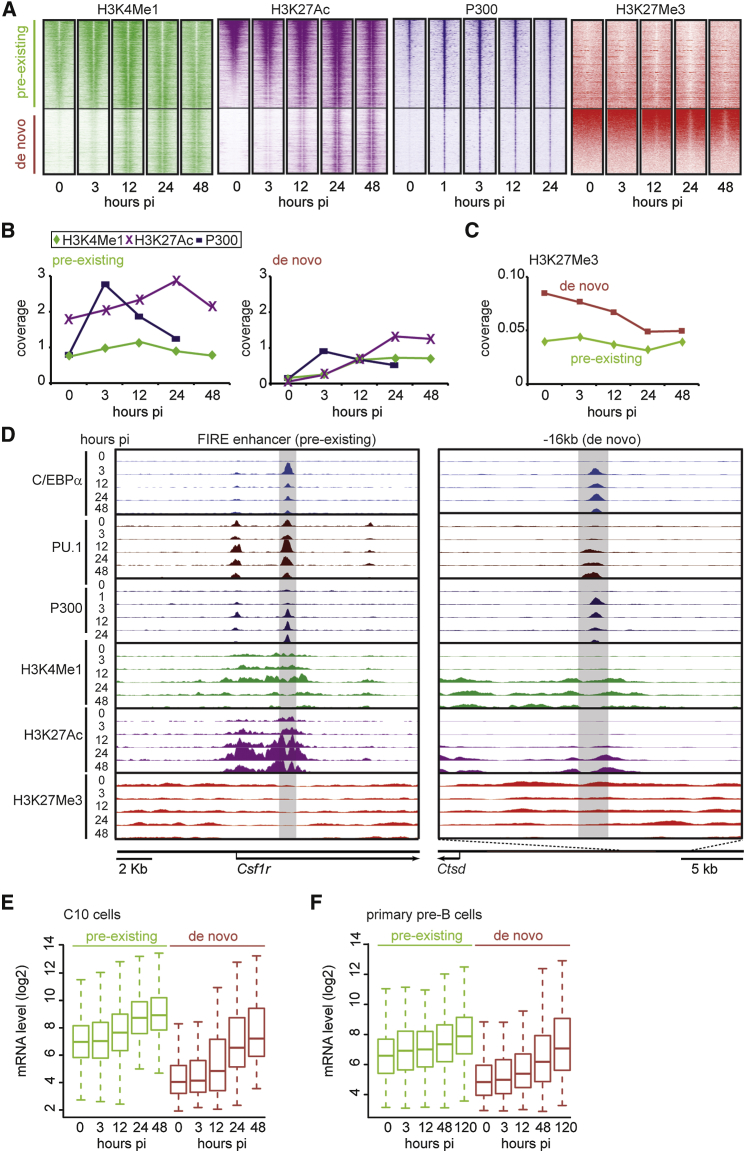
Kinetics of Histone Marks, P300 Binding, and Gene Expression Associated with Myeloid Enhancers (A) Heatmaps visualizing H3K4Me1, H3K27Ac, P300 binding, and H3K27Me3 at pre-existing and de novo enhancers at different hpi of induced C10 cells. Window, 6,000 bp; bin, 100. See also Figure S5A. (B) Quantification of H3K4Me1, H3K27Ac, and P300, as in (A). Bins with the highest coverage are shown. (C) Quantification of H3K27Me3, as in (A), except values at the center position are shown. See also Figure S5B. (D) Screenshots of selected enhancers showing C/EBPα, PU.1, P300, H3K4Me1, H3K27Ac, and H3K27Me3 profiles in C10 cells. See also Figure S5C. (E and F) Distribution of mRNA levels of upregulated genes nearest to either pre-existing (n = 318) or de novo (n = 103) enhancers during transdifferentiation of C10 cells and primary pre-B cells is shown. See also Figures S5D and S5E.

To determine whether the epigenetic status of the two enhancer types in pre-B cells influences their subsequent activation kinetics, we analyzed enrichment levels of H3K4Me1, H3K27Ac, P300, and H3K27Me3 during transdifferentiation. As judged by P300 and H3K27Ac, pre-existing enhancers became hyper-activated, albeit mostly in a transient manner. In contrast, de novo enhancers became gradually activated, starting from background levels (Figures 5A, 5B, and S5A). Both enhancer types followed a similar sequence of enhancer mark acquisition, consisting in P300 binding followed by H3K4Me1 and H3K27Ac decoration (Figure 5B). In contrast, the repressive H3K27Me3 decreased, predominantly on de novo enhancers (Figures 5C and S5B). These findings are illustrated for the pre-existing 3-kb FIRE enhancer of the *Csf1r* gene and the de novo −16-kb enhancer of *Ctsd* (Figure 5D). Additional examples are shown in Figure S5C.

To determine how the two types of prospective myeloid enhancers modulate the upregulation kinetics of adjacent genes, we interrogated gene expression data from C10 cells and primary pre-B cells induced to transdifferentiate (Bussmann et al., 2009; Di Tullio et al., 2011). Pre-existing enhancer-associated genes started from low expression levels and became gradually upregulated ∼4-fold, while de novo enhancer-associated genes started from background levels and were upregulated ∼9-fold (Figures 5E, 5F, S5D, and S5E).

In sum, the C/EBPα and PU.1 binding order determines the activation kinetics of targeted enhancers, with pre-existing enhancers becoming activated gradually from detectable base levels and de novo enhancers becoming activated more steeply and with a delay. These differences also are reflected in the activation kinetics of adjacent genes.

### Pre-existing and De Novo Enhancers Are in an Active State in Distinct Hematopoietic Cell Types

**Figure 6:**
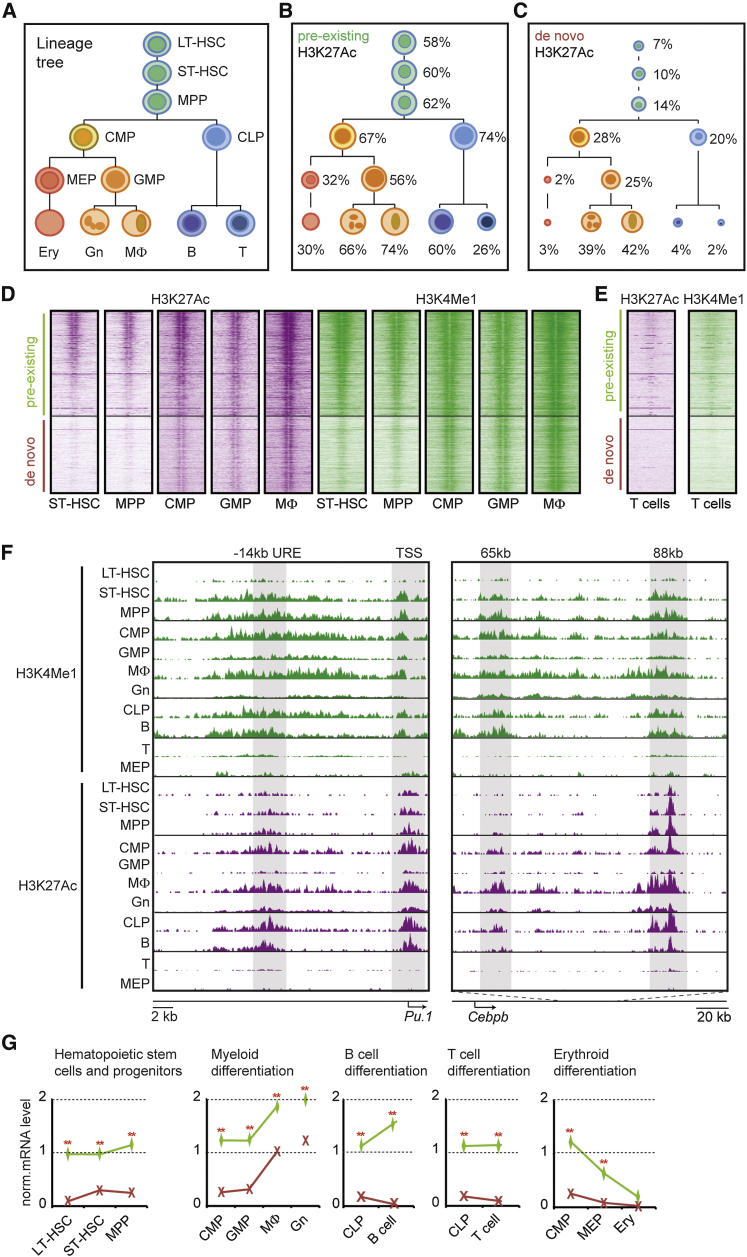
Distribution of Active Myeloid Enhancers during Hematopoietic Differentiation (A) Cartoon depicting blood cell lineage specification. (B and C) Percentage of pre-existing and de novo macrophage enhancers intersecting with enhancers decorated with H3K27Ac in different hematopoietic progenitors and differentiated cell types. The size of the circles relative to circles in (A) indicates the percentage of representation. See also Figures S6A and S6B. (D) Heatmaps visualizing H3K27Ac and H3K4Me1 decoration at pre-existing and de novo enhancers during myeloid differentiation. Window, 6,000 bp; bin, 100. (E) As in (D), but for T cells. (F) Screenshots of H3K4Me1 and H3K27Ac profiles at selected C/EBPα-bound enhancers of *Pu.1* and *Cebpb* in the indicated hematopoietic cell types. (G) Median mRNA levels of genes nearest either pre-existing (green lines; n = 318) or de novo (red lines; n = 103) enhancers in different hematopoietic stem/progenitors and differentiated cells. Statistical analysis by Wilcoxon rank-sum test, ^∗∗^p < 0.001. See also Figures S6C and S6D.

Are the pre-existing and de novo myeloid enhancers identified during transdifferentiation relevant for normal hematopoietic differentiation? To study this we determined their activation state in various types of immature and mature hematopoietic cells and interrogated expression data of associated genes during hematopoiesis (Lara-Astiaso et al., 2014; see Figure 6A for the hematopoietic lineage tree and nomenclature used). Surprisingly, ∼58% of pre-existing enhancers already were active (i.e., marked by H3K27Ac) in long-term hematopoietic stem cells (LT-HSCs), and their proportion further increased in common myeloid and lymphoid progenitors (CMPs and CLPs, respectively), reaching ∼66% and ∼74% in terminally differentiated granulocytes (Gns) and MΦ, respectively (Figures 6B and S6A). Moreover, a substantial fraction of pre-existing enhancers remained active in B cells (60%) but decreased in megakaryocyte-erythroid progenitors (MEPs), erythroid cells (Erys), and T cells (∼30%) (Figures 6B and S6A). In contrast, activated de novo enhancers were essentially restricted to the myeloid compartment with 25%–28% being decorated with H3K27Ac in CMPs and GMPs and ∼40% in Gns and MΦs, while HSCs and multipotent progenitors (MPPs) showed lower percentages (7% and 14%) and MEPs, Erys, and B and T cells were essentially negative (Figures 6C and S6A). Similar trends were observed for pre-existing and de novo enhancers marked with H3K4Me1 (Figure S6B). Heatmaps of the two enhancer types during the transition from short-term hematopoietic stem cells (ST-HSCs) to macrophages (Figure 6D) were remarkably similar to those of pre-B cells transdifferentiating into macrophages (see Figure 5A). In contrast, the two enhancer types were not activated in T cells (Figure 6E).

These findings are illustrated for the pre-existing −14-kb URE enhancer of *Pu.1* and the 88-kb putative enhancer of *Cebpb* (Figure 6F), which are bound by PU.1 (Figure 1G). A de novo enhancer is exemplified by the 65-kb enhancer of *Cebpb* (Figure 6F). Strikingly, mRNA levels of genes associated with pre-existing and de novo enhancers reflected enhancer activity during hematopoiesis using two independently derived datasets analyzed by either RNA-seq or expression arrays (Figures 6G, S6C, and S6D; Lara-Astiaso et al., 2014; Di Tullio et al., 2011). The arrays also showed that in normal macrophages the expression levels of genes associated with the two enhancer types nearly converged (Figure S6D).

In conclusion, our data show that the majority of pre-existing enhancers targeted by C/EBPα during transdifferentiation are broadly active in hematopoietic stem cells, progenitors, and B cells, whereas de novo enhancers are largely restricted to the myeloid compartment.

### The Activity of the Two Enhancer Types Reflect *Pu.1*, *Cebpa*, and *Cebpb* Expression during Hematopoiesis

**Figure 7:**
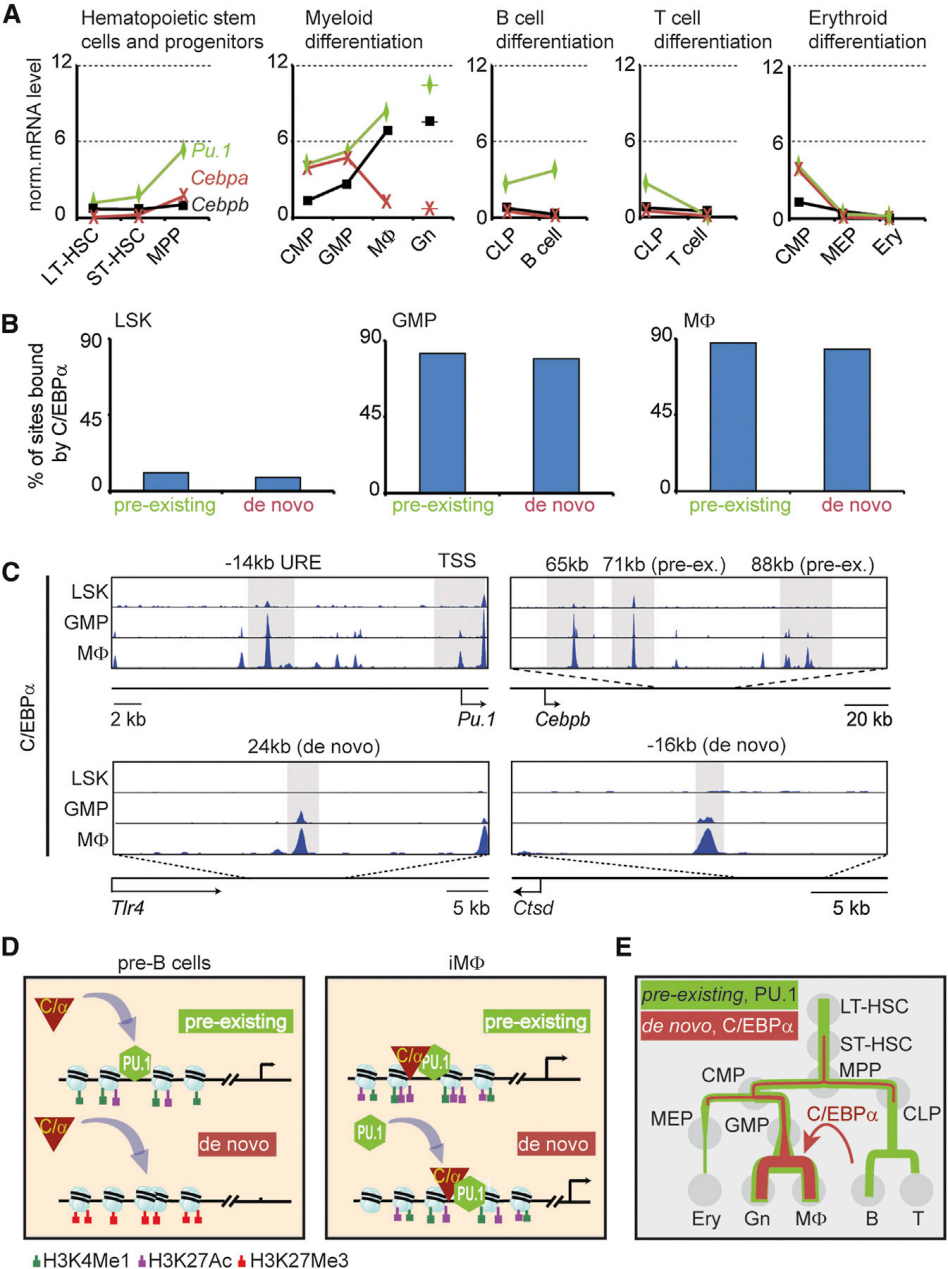
Expression Kinetics of Genes Associated with Pre-existing and De Novo Enhancers during Hematopoiesis (A) mRNA levels of *Pu.1*, *Cebpa*, and *Cebpb* in different hematopoietic stem/progenitor cell types, based on RNA-seq data (Lara-Astiaso et al., 2014). (B) Percentages of pre-existing or de novo enhancers bound by C/EBPα in early hematopoietic progenitors (Lin- Sca-1+ Kit+, LSK cells), GMPs, or primary MΦ. (C) Screenshots of C/EBPα bound to selected enhancers of *Pu.1*, *Cebpb*, *Tlr4*, and *Ctsd* in the indicated hematopoietic cell types. Pre-ex., pre-existing. (D) Pre-existing and de novo myeloid enhancers in pre-B cells and iMΦ, showing PU.1 occupancy, binding sites targeted by incoming C/EBPα (curved arrows), enhancer states, and gene expression. Nucleosomes are indicated by light blue balls. (E) Artist’s rendering of the trajectory of activated pre-existing enhancers within the hematopoietic lineage tree (in green) and de novo enhancers (in red). The arrow depicts how C/EBPα short circuits the two trajectories when expressed in pre-B cells.

How are the two enhancer types observed during C/EBPα-induced transdifferentiation controlled during normal hematopoiesis? To study this we analyzed the expression of *Pu.1*, *Cebpa*, and *Cebpb* during hematopoietic differentiation. *Pu.1* expression was found to closely correlate with that of pre-existing enhancers, *Pu.1* being broadly expressed in stem and progenitor cells and weakly in T cells, MEPs, and Erys (Figure 7A). Similar expression patterns were observed at the protein level with PU.1 reporter mice (Back et al., 2005). In turn, *Cebpa* was expressed mostly in the myeloid compartment where its levels were highest in GMPs (Figure 7A; Wölfler et al., 2010), in agreement with the fact that mice lacking C/EBPα do not develop GMPs (Zhang et al., 2004). In contrast, *Cebpb* expression reached highest levels in macrophages and Gns (Figure 7A), suggesting that C/EBPβ takes over the role of C/EBPα in terminally differentiated myeloid cells. This interpretation agrees with the fact that macrophages from C/EBPβ-knockout mice have functional defects (Chen et al., 1997; Tanaka et al., 1995).

To test whether the *Cebpa* expression pattern reflects its binding specificity in the hematopoietic system, we analyzed the C/EBPα-binding sites identified in pre-existing and de novo enhancers in stem and progenitors cells and GMPs, as previously reported (Hasemann et al., 2014). Strikingly, <10% of prospective myeloid enhancers were bound by C/EBPα in the progenitors, while ∼80% of the sites were bound in GMPs and in primary macrophages (Figure 7B). C/EBPα binding in progenitor cells and primary MΦ is illustrated for the *PU.1* and *Cebpb* genes (Figure 7C) as well as for the *Tlr4* and *Ctsd* genes.

Together, our observations indicate that, within the hematopoietic system, the combination of *Pu.1*, *Cebpa*, and *Cebpb* determines the activity of the two types of prospective macrophage enhancers and, hence, the expression of adjacent genes in a manner that recapitulates C/EBPα-induced transdifferentiation.

## Discussion

Our study of C/EBPα-induced pre-B-cell-to-macrophage transdifferentiation has revealed two types of prospective myeloid enhancers that are activated by C/EBPα. Pre-existing enhancers in pre-B cells are decorated with active enhancer marks and bound in their majority by PU.1, while de novo enhancers are free of enhancer activation marks and free of PU.1; C/EBPα simultaneously hyper-activates pre-existing enhancers and newly activates de novo enhancers (summarized in Figure 7D). These enhancers drive a substantial part of the gene repertoire required for the formation of functional macrophages. Strikingly, we also observed a similar synergy between pre-existing and de novo enhancers during myeloid lineage specification during normal hematopoiesis (Figure 7E).

The finding that pre-existing-type myeloid enhancers drive low-level expression of adjacent myeloid-restricted genes in early hematopoietic progenitors provides a mechanistic explanation for the phenomenon dubbed “lineage priming” (Hu et al., 1997). The observed expression of the myeloid markers lysozyme and CSF-1 receptor in hematopoietic stem cells (Mossadegh-Keller et al., 2013; Ye et al., 2003) supports this interpretation. The following observations indicate that PU.1 is a key component in the generation of pre-existing myeloid enhancers: (1) most pre-existing enhancers are bound by the factor; (2) PU.1 is expressed in stem and progenitor cells, but downregulated in T cells and Erys (Back et al., 2005; Lara-Astiaso et al., 2014; Nutt et al., 2005), and this strongly correlates with the distribution of pre-existing myeloid enhancers and the expression of nearby genes; and (3) overexpression of PU.1 in fibroblasts partially activates myeloid genes associated with pre-existing enhancers (Figure 2G) and C/EBPα further enhances their expression, while C/EBPα alone has no effect (Feng et al., 2008).

However, it is likely that, in addition to PU.1, other TFs participate in the initiation of the establishment of pre-existing enhancers and the activation of de novo enhancers. Thus, C/EBPα sites also were enriched for the RUNX motif, in line with the finding that during myelopoiesis Runx1 binds transiently to the URE element of the *Pu.1* gene to establish open chromatin, permitting the binding of PU.1 (Hoogenkamp et al., 2009). In addition, it is possible that the *Fos* gene acts as a downstream effector, as it is directly regulated by C/EBPα and we observed enrichment of AP-1 motifs in C/EBP-bound sites where it might co-operate with C/EBPs.

The weakly active pre-existing myeloid enhancers in hematopoietic progenitors appear to be in a stand-by state that can be fully activated by changes in the bone marrow microenvironment either during development or in adult life, such as after infections with pathogens. These signals may in turn increase the levels of PU.1, C/EBPα, and C/EBPβ expression. Thus, for example, bacteria or inflammatory stimuli can upregulate *Pu.1* expression in hematopoietic stem cells through the activation of M-CSF, a cytokine that in turn activates the CSF-1 receptor (Mossadegh-Keller et al., 2013). In addition, the yeast *Candida albicans* can induce emergency granulopoiesis in hematopoietic progenitors through upregulation of C/EBPβ (Hirai et al., 2006). Therefore, the ectopic expression of C/EBPα/β to induce transdifferentiation of pre-B cells might mimic processes that are normally triggered in hematopoietic progenitors by developmental cues or pathogens.

Surprisingly, a subset of pre-existing enhancers appears to be bi-functional. In B cells this subset is bound by the B cell TF Ebf1, typically in combination with PU.1, resulting in low-level expression. Binding of C/EBPα further activates these genes, raising the possibility that their products are themselves bi-functional. The *Cebpb* gene illustrates this scenario as its putative 88-kb upstream enhancer is bound by Ebf1, which is eventually replaced by C/EBPα during the conversion into myeloid cells. In addition the factor is required for the function of both B cells and macrophages (Chen et al., 1997; Tanaka et al., 1995). However, whether the 88-kb site is the physiologically most relevant *Cebpb* enhancer is unknown.

Previous work on TF combinations that induce cell fate conversions have postulated two alternative models as follows: (1) a symmetric collaboration between various TFs acting as pioneer factors, exemplified by Oct4, Sox2, and Klf4 that act during iPSC reprogramming (Soufi et al., 2012); and (2) a hierarchical model, exemplified by Ascl1 acting as a pioneer for the subsequent binding of Brn2 and Myt1l during induced neuronal transdifferentiation (Wapinski et al., 2013). Here we propose a mixed model, where the key lineage-instructive factors exert dual roles as both pioneer and secondary factors. The conclusion that C/EBPα can act as a pioneer factor is based on the observation that it binds to chromatin regions free of activating histone marks and to a nucleosome-dense region within de novo enhancers, agreeing with the reported pioneer activity of C/EBPβ (Siersbæk et al., 2011). It is possible that PU.1 also can act as a pioneer factor, as it is one of the earliest lineage-instructive factors expressed in the hematopoietic system (Dzierzak and Speck, 2008), and on its own can induce the expression of myeloid genes in non-hematopoietic cells.

In conclusion, our work revealed that the collaboration between an exogenous and an endogenous lineage-instructive TF (C/EBPα and PU.1) leads to the activation of pre-existing and de novo myeloid enhancers during transdifferentiation, resulting in macrophage differentiation. Interestingly, this mechanism recapitulates the way endogenous C/EBP factors and PU.1 collaborate to induce myeloid differentiation during normal hematopoiesis. It will be interesting to determine whether conversions of other cell types driven by TFs likewise recapitulate developmental processes that result from the superimposition of complementary enhancer types.

## Experimental Procedures

### Cell Culture, Retroviruses, and shRNA Constructs

The origin of the HAFTL pre-B cell line, its derivatives C10 (C/EBPαER-GFP) and C11 (C/EBPαER-hCD4), and induction of transdifferentiation (treatment with 100 uM β-est and grown in the presence of 10 nM Il-3 and 10 nM CSF-1) have been described previously (Bussmann et al., 2009; Xie et al., 2004). The shC/EBPβ-KD07 directed to the ORF of *Cebpb* was purchased from Sigma (Mission shRNA System) in a pLKO.1-puro lentiviral backbone. An shRNA against PU.1 cloned into LMP-GFP virus (Open Biosystems) was a gift from Dr. M. Sieweke (Sarrazin et al., 2009). The 3T3 cell culture conditions and the PU.1-GFP construct have been described previously (Feng et al., 2008). Phagocytosis of yeast was performed as described previously by Rapino et al. (2013). To test for statistical differences of C/EBPα binding after knockdown of PU.1, we applied the Student’s t test, one-tailed, alpha level (0.05).

### FACS

FACS experiments were performed as described previously (Bussmann et al., 2009) using conjugated antibodies against Cd19 (550992) and Cd11b (552850) and combined with blocking antibody (553142) from BD Pharmingen. Unstained cells or an isotype control antibody (553932, BD Pharmingen) were used as a negative control.

### ChIP, ChIP-Seq, and MNase-Seq Experiments

ChIP experiments were performed as described previously (van Oevelen et al., 2008). DNA libraries were prepared using Illumina’s reagents and instructions. Nucleosome positioning was determined by MNase digestion using a modification of a published method (Cappabianca et al., 1999). All libraries were sequenced on the Illumina GA IIx or Hiseq2000 sequencer.

### Processing of ChIP-Seq and MNase-Seq Data

High-throughput Illumina sequencing data were base-called using the Illumina pipeline, and sequencing reads were aligned to the mouse genome (mm9) using either the Illumina Eland alignment tool or Bowtie (Langmead et al., 2009) without mismatches. Aligned sequences were filtered to remove identical sequence tags and sequence tags not aligning uniquely to the mouse genome. To detect enriched regions, we used HOMER (http://homer.salk.edu/homer/ngs/index.html) (Heinz et al., 2010; and see Table S1). See the Supplemental Experimental Procedures for further details. To test for statistical differences in the level or reduction of coverage between sets of regions, we applied the Wilcoxon rank-sum test, two-tailed, alpha level (0.05).

### Binding Site Annotation, Motif Analysis, and Gene Expression

Position of non-redundant regions relative to TSS of nearest gene (RefSeq mm9) was based on center position and calculated by in-house Perl scripts. For a subset of genes, the median expression level was calculated, and, to test for statistical differences in gene expression levels between sets of genes, we applied the Wilcoxon rank-sum test, two-tailed, alpha level (0.05). Gene expression values in hematopoietic cells (Lara-Astiaso et al., 2014) were normalized by dividing each presented mRNA value by the average mRNA of all listed genes per cell type. To annotate genes for enrichment of GO terms, we employed David with standard settings (Huang da et al., 2009). Motif discovery within selected regions was performed using HOMER (Heinz et al., 2010).

### Gene Expression Analyses by qRT-PCR

To analyze mRNA levels of selected genes in either C10 cells induced with β-est or 3T3 cells overexpressing PU.1, we extracted RNA using trizol and reverse transcribed it with GeneAmp RNA PCR (Applied Biosystems). SybrGreen PCR Master Mix (Applied Biosystems) was used for amplification and detection of cDNAs, and PCR reactions were carried out with the AB7900HT detection system (Applied Biosystems). To test for statistical differences in mRNA levels, we applied the Student’s t test, one-tailed, alpha level (0.05).

## Accession Numbers

The accession numbers for the ChIP-seq data reported in this paper are GEO: GSE53173, GSE53362, and GSE53460 for chromatin marks, factor binding, and MNase, respectively.

## Supplemental Information

Document S1. Supplemental Experimental Procedures and Figures S1–S6

Table S1. Summary of Peak Calling Statistics of TFs and Chromatin Marks by HOMER

Table S2. Peak Characteristics, Annotation, and Gene Expression of Nearest Genes of C/EBPα-Enriched Regions in Induced Macrophages

Table S3. Genomic Coordinates of University of California, Santa Cruz, Screenshot Examples and Tested Loci for ChIP, RefSeq mm9/10

Table S4. Primer Sequences

Movie S1. Transdifferentiation in Action The video shows a culture of C10 cells labeled with GFP surrounded by red fluorescent yeast (*Candida albicans*). As the TF C/EBPα is activated within the B cells, these aggregate and turn into macrophages that ingest the yeast, so that 51 hr after activation all pathogens were eaten. The microscope used for the acquisition was a Zeiss Cell Observer HS.

Document S2. Article plus Supplemental Information
