# Supplementary material for: C/EBPα Activates Pre-existing and De Novo Macrophage Enhancers during Induced Pre-B Cell Transdifferentiation and Myelopoiesis
Source: Stem Cell Reports. 2015 Jul 30;5(2):232–47. doi: 10.1016/j.stemcr.2015.06.007 (PMC4618662; doi:10.1016/j.stemcr.2015.06.007)
Supplement: Document S1. Supplemental Experimental Procedures and Figures S1–S6 [file mmc1.pdf]

Stem Cell Reports
Supplemental Information

C/EBPα Activates Pre-existing and De Novo Macrophage Enhancers during Induced Pre-B Cell Transdifferentiation and Myelopoiesis

Chris van Oevelen, Samuel Collombet, Guillermo Vicent, Maarten Hoogenkamp, Cyrille Lepoivre, Aimee Badeaux, Lars Bussmann, Jose Luis Sardina, Denis Thieffry, Miguel Beato, Yang Shi, Constanze Bonifer, and Thomas Graf

Figure S1

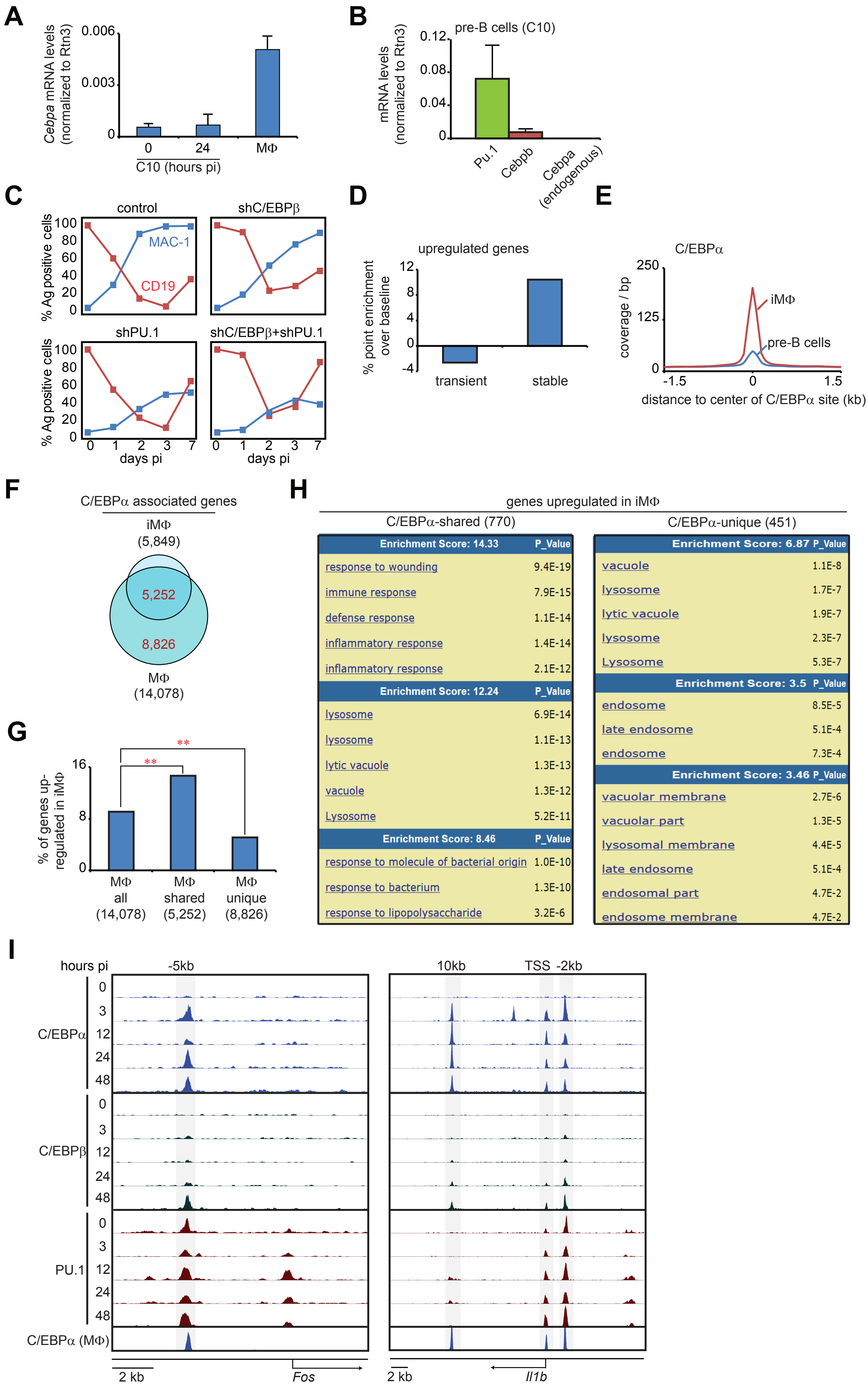
A
Cebpa mRNA levels (normalized to Rtn3)
0.006
0.003
0
0
24
MΦ
C10 (hours pi)
B
pre-B cells (C10)
mRNA levels (normalized to Rtn3)
0.12
0.08
0.04
0
Pu.1
Cebpb
Cebpa (endogenous)
C
control
shC/EBPβ
shPU.1
shC/EBPβ+shPU.1
% Ag positive cells
MAC-1
CD19
100
80
60
40
20
0
0 1 2 3 7
days pi
D
upregulated genes
% point enrichment over baseline
12
8
4
0
-4
transient
stable
E
C/EBPα
coverage / bp
250
125
0
iMΦ
pre-B cells
-1.5
0
1.5
distance to center of C/EBPα site (kb)
F
C/EBPα associated genes
iMΦ
(5,849)
5,252
8,826
MΦ
(14,078)
G
% of genes up-regulated in iMΦ
16
8
0
**
**
MΦ all (14,078)
MΦ shared (5,252)
MΦ unique (8,826)
H
genes upregulated in iMΦ
C/EBPα-shared (770)
Enrichment Score: 14.33 P_Value
response to wounding 9.4E-19
immune response 7.9E-15
defense response 1.1E-14
inflammatory response 1.4E-14
inflammatory response 2.1E-12
Enrichment Score: 12.24 P_Value
lysosome 6.9E-14
lysosome 1.1E-13
lytic vacuole 1.3E-13
vacuole 1.3E-12
Lysosome 5.2E-11
Enrichment Score: 8.46 P_Value
response to molecule of bacterial origin 1.0E-10
response to bacterium 1.3E-10
response to lipopolysaccharide 3.2E-6
C/EBPα-unique (451)
Enrichment Score: 6.87 P_Value
vacuole 1.1E-8
lysosome 1.7E-7
lytic vacuole 1.9E-7
lysosome 2.3E-7
Lysosome 5.3E-7
Enrichment Score: 3.5 P_Value
endosome 8.5E-5
late endosome 5.1E-4
endosome 7.3E-4
Enrichment Score: 3.46 P_Value
vacuolar membrane 2.7E-6
vacuolar part 1.3E-5
lysosomal membrane 4.4E-5
late endosome 5.1E-4
endosomal part 4.7E-2
endosome membrane 4.7E-2
I
hours pi
-5kb
10kb
TSS -2kb
C/EBPα
C/EBPβ
PU.1
0
3
12
24
48
C/EBPα (MΦ)
2 kb
Fos
2 kb
Il1b

Figure S2

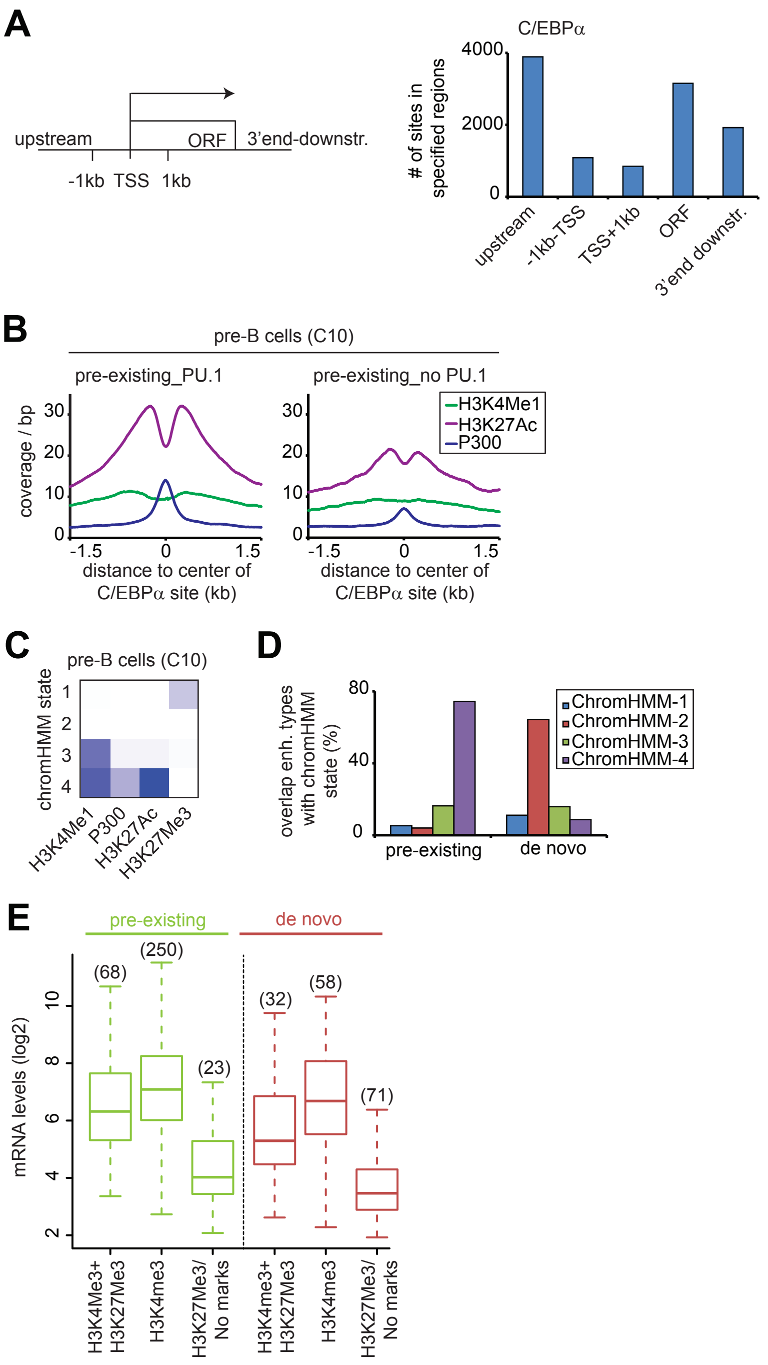
A
upstream
ORF
3'end-downstr.
-1kb TSS 1kb
C/EBPα
# of sites in specified regions
4000
2000
0
upstream
-1kb-TSS
TSS+1kb
ORF
3'end downstr.
B
pre-B cells (C10)
pre-existing_PU.1
pre-existing_no PU.1
coverage / bp
H3K4Me1
H3K27Ac
P300
distance to center of C/EBPα site (kb)
C
pre-B cells (C10)
chromHMM state
H3K4Me1
P300
H3K27Ac
H3K27Me3
D
overlap enh. types with chromHMM state (%)
ChromHMM-1
ChromHMM-2
ChromHMM-3
ChromHMM-4
pre-existing
de novo
E
pre-existing
de novo
mRNA levels (log2)
(68)
(250)
(23)
(32)
(58)
(71)
H3K4Me3+ H3K27Me3
H3K4me3
H3K27Me3/ No marks
H3K4me3+ H3K27Me3

Figure S3

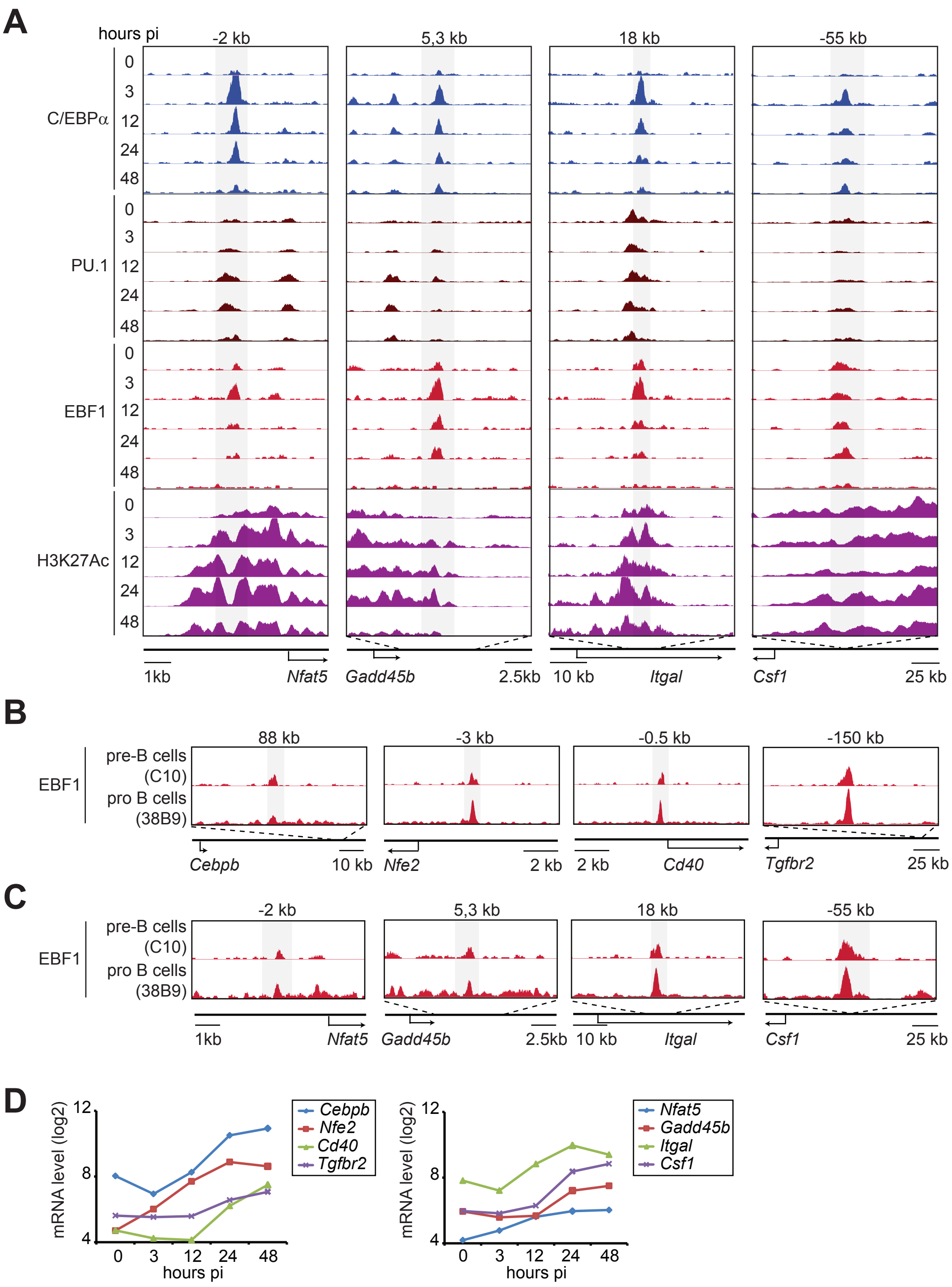

A
hours pi
-2 kb
5,3 kb
18 kb
-55 kb
C/EBPα
PU.1
EBF1
H3K27Ac
0
3
12
24
48
1kb
Nfat5
Gadd45b
2.5kb
10 kb
Itgal
Csf1
25 kb
B
EBF1
pre-B cells (C10)
pro B cells (38B9)
88 kb
-3 kb
-0.5 kb
-150 kb
Cebpb
10 kb
Nfe2
2 kb
2 kb
Cd40
Tgfbr2
25 kb
C
EBF1
pre-B cells (C10)
pro B cells (38B9)
-2 kb
5,3 kb
18 kb
-55 kb
1kb
Nfat5
Gadd45b
2.5kb
10 kb
Itgal
Csf1
25 kb
D
mRNA level (log2)
12
8
4
0
3
12
24
48
hours pi
Cebpb
Nfe2
Cd40
Tgfbr2
Nfat5
Gadd45b
Itgal
Csf1

Figure S4

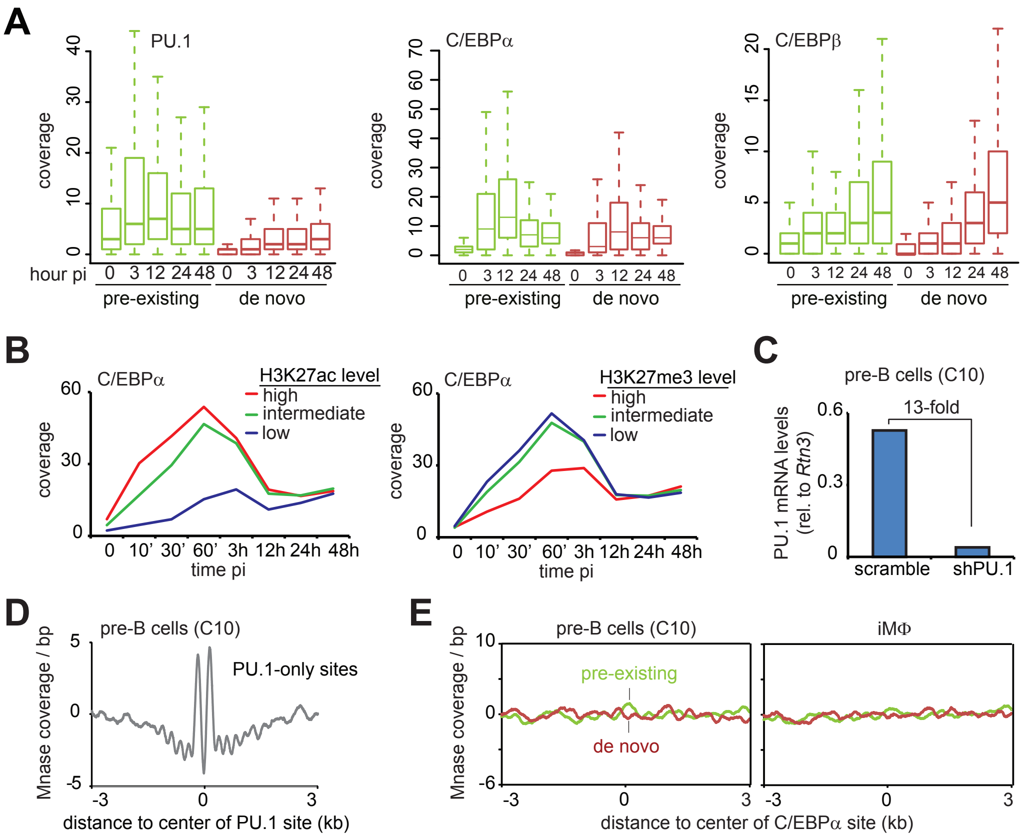
A
PU.1
C/EBPα
C/EBPβ
coverage
hour pi
0 3 12 24 48 0 3 12 24 48
pre-existing
de novo
B
C/EBPα
H3K27ac level
high
intermediate
low
H3K27me3 level
time pi
0 10' 30' 60' 3h 12h 24h 48h
C
pre-B cells (C10)
13-fold
PU.1 mRNA levels
(rel. to Rtn3)
scramble
shPU.1
D
pre-B cells (C10)
PU.1-only sites
Mnase coverage / bp
distance to center of PU.1 site (kb)
E
pre-B cells (C10)
iMΦ
pre-existing
de novo
distance to center of C/EBPα site (kb)

Figure S5

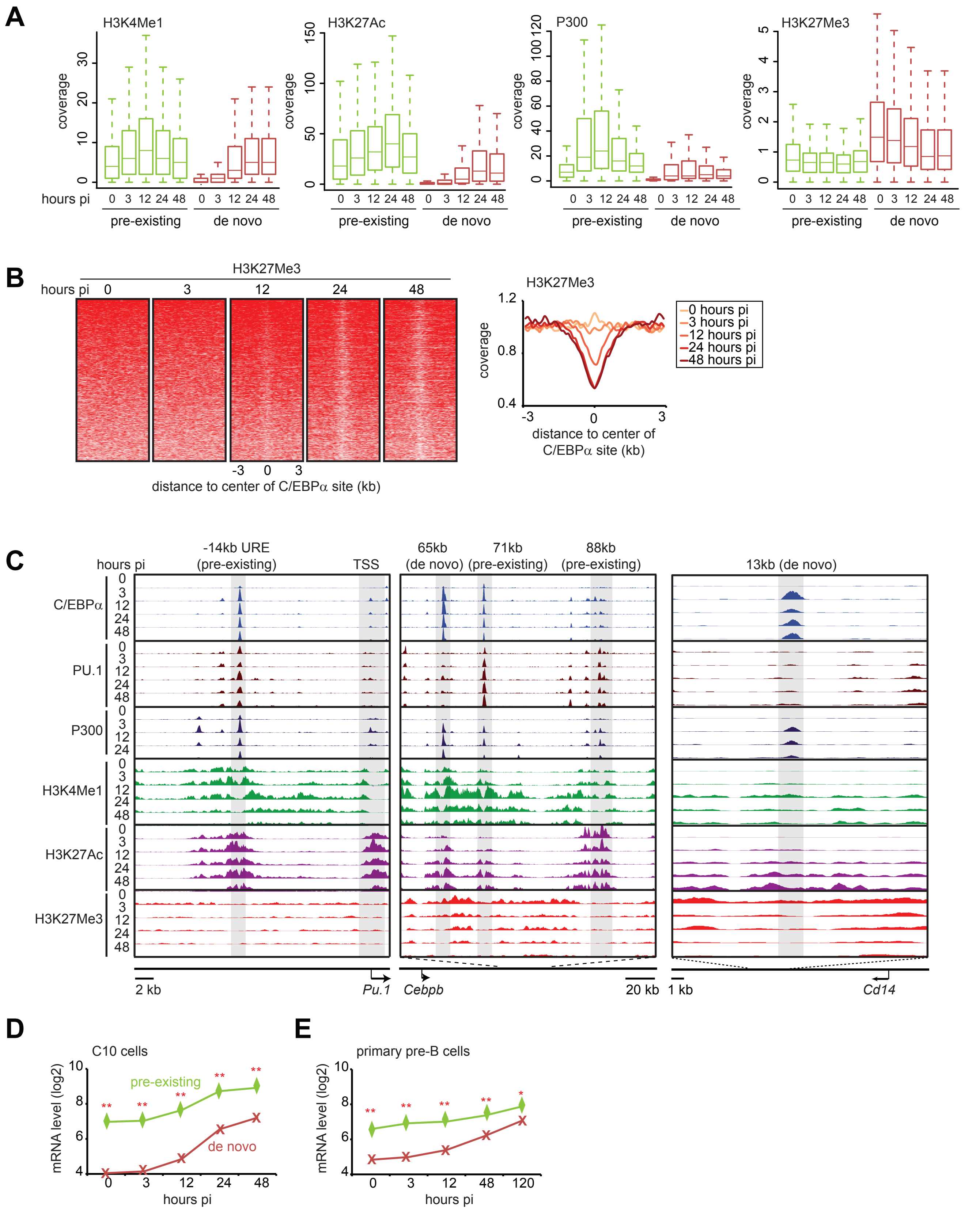
A
H3K4Me1
H3K27Ac
P300
H3K27Me3
coverage
hours pi
0 3 12 24 48
pre-existing
de novo
B
H3K27Me3
hours pi 0 3 12 24 48
-3 0 3
distance to center of C/EBPα site (kb)
coverage
0 hours pi
3 hours pi
12 hours pi
24 hours pi
48 hours pi
distance to center of C/EBPα site (kb)
C
hours pi
-14kb URE (pre-existing)
TSS
65kb (de novo)
71kb (pre-existing)
88kb (pre-existing)
13kb (de novo)
C/EBPα
PU.1
P300
H3K4Me1
H3K27Ac
H3K27Me3
2 kb
Pu.1
Cebpb
20 kb
1 kb
Cd14
D
C10 cells
mRNA level (log2)
pre-existing
de novo
hours pi
E
primary pre-B cells
mRNA level (log2)
hours pi

Figure S6

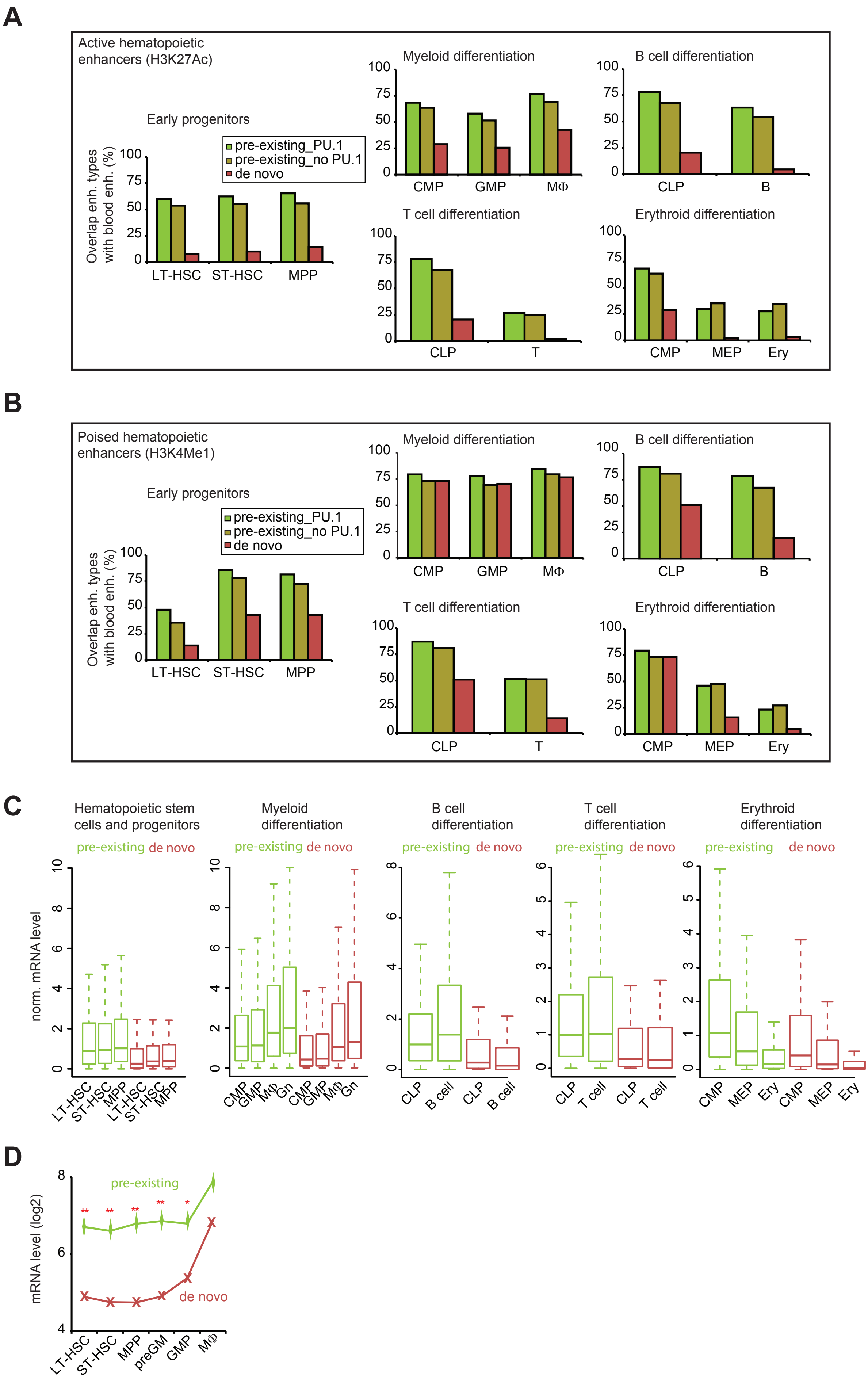
A
Active hematopoietic enhancers (H3K27Ac)
Early progenitors
pre-existing_PU.1
pre-existing_no PU.1
de novo
Overlap enh. types with blood enh. (%)
LT-HSC
ST-HSC
MPP
Myeloid differentiation
CMP
GMP
MΦ
B cell differentiation
CLP
B
T cell differentiation
CLP
T
Erythroid differentiation
CMP
MEP
Ery
B
Poised hematopoietic enhancers (H3K4Me1)
Early progenitors
pre-existing_PU.1
pre-existing_no PU.1
de novo
Overlap enh. types with blood enh. (%)
LT-HSC
ST-HSC
MPP
Myeloid differentiation
B cell differentiation
T cell differentiation
Erythroid differentiation
C
Hematopoietic stem cells and progenitors
Myeloid differentiation
B cell differentiation
T cell differentiation
Erythroid differentiation
pre-existing
de novo
norm. mRNA level
D
mRNA level (log2)
pre-existing
de novo
LT-HSC
ST-HSC
MPP
preGM
GMP
MΦ

Supplemental Figures

Supplemental Figure S1. PU.1 and C/EBPβ are required for B cell to macrophage transdifferentiation and characterization of C/EBPα binding sites.

(**A+B**) Expression of C/EBPα (exogenous and endogenous) in C10 cells (0 and 24 hours pi) and primary MΦ (A) and *Pu.1*, *Cebpb* and *Cebpa* (endogenous) in uninduced pre B cells (B) as measured by qRT-PCR (technical triplicates) normalized to the mRNA levels of *Rtn3*. Data are represented as mean ± SEM. (**C**) Percentage of CD19 or MAC-1 positive cells (antigen: Ag) was determined by FACS after ablation of C/EBPβ, PU.1 or both. (**D**) C/EBPα sites (n=54,198) were grouped based on either transient or stable C/EBPα binding (stable binding: up to 48 hours pi). For each group the number of sites nearest upregulated genes was calculated, compared to all sites (baseline) and expressed as percent point difference. **(E)** C/EBPα binding in uninduced B cells and iMΦ (48 hours pi). Center of binding=0, Window= 3kb, bin=1 (**F**) Venn diagram showing the intersection of C/EBPα associated genes in iMΦ (n=5,849) and primary macrophages (MΦ, n=14,078) by nearest gene approach (**G**) Percentage of all, shared or unique C/EBPα associated genes that become upregulated during transdifferentiation of primary B cells. Statistical analysis by hypogeometric distribution; two asterixes p<0.001. **(H)** Functional annotation analysis of shared (n=770, left panel) or unique (n=451, right panel) C/EBPα associated genes which become upregulated during transdifferentiation by DAVID. First three enrichment clusters of each group is shown (**I**) Screenshots of C/EBPα, C/EBPβ and PU.1 binding at enhancer regions associated with the *Fos* and *Il1b* genes in C10 cells induced for different times and C/EBPα binding in primary MΦ. Arrows indicate TSS and direction of transcription.

Supplemental Figure S2. Chromatin state maps in uninduced B cells

(**A**) Genomic distribution of stable C/EBPα binding events (n=10,849) relative to the transcription start site (TSS) in induced MΦ. ORF: open reading frame (**B**) Coverage of H3K4Me1, H3K27Ac and P300 around *pre-existing* sites bound or not bound by PU.1. Window= 3 kb, bin: 1 bp (**C**) Chromatin

state map in uninduced B cells by ChromHMM. Number of states was pre-set to 4. (**D**) Comparison of *pre-existing* or *de novo* enhancers with ChromHMM state maps. (**E**) Distribution of mRNA levels of genes nearest *pre-existing* or *de novo* enhancers in uninduced C10 cells. Genes were subdivided based on promoter activity. Number of genes in brackets.

Supplemental Figure S3. Binding of the B cell transcription factor EBF1 to myeloid enhancers.

(**A**) Screenshots of C/EBPα, PU.1, EBF1 and H3K27Ac ChIPseq profiles at selected enhancer regions in C10 cells. (**B+C**) Screenshots of EBF1 binding at enhancer regions as shown in Figure 3F and panel A in uninduced C10 cells and a pro-B cell line (38B9). (**D**) Gene expression kinetics of genes associated with prospective myeloid enhancers bound by EBF1 (see Fig. 3F and panel A) by microarray.

Supplemental Figure S4. C/EBPα and PU.1 binding kinetics and nuclease accessibility at macrophage enhancers during transdifferentiation.

(**A**) Distribution of PU.1, C/EBPα and C/EBPβ coverage values of *pre-existing* or *de novo* enhancers at indicated times after induction. Values of the center position are shown. (**B**) Kinetics of C/EBPα (at center position) of *pre-existing* enhancers grouped by H3K27Ac levels or *de novo* enhancers grouped by H3K27Me3 levels at indicated times post induction. 10, 30 and 60 minutes, h: hours (**C**) *Pu.1* mRNA levels after ablation of PU.1 by shRNA in C10 cells as measured by qRT-PCR (technical triplicates) normalized to the mRNA levels of the *Rtn3* gene. (**D**) Average profile of MNase resistant DNA at PU.1 sites devoid of C/EBPα and C/EBPβ binding (n=8,765) in pre-B cells. Profiles were generated as in Figs. 4F,G. (**E**) Average profile of MNase resistant DNA at random positions in the genome. Profiles were generated as in Figs. 4F,G.

Supplemental Figure S5. Chromatin kinetics at macrophage enhancers during transdifferentiation

(**A**) Distribution of H3K4Me1, H3K27Ac, P300, and H3K27Me3 coverage values at center position of *pre-existing* or *de novo* enhancers at indicated times after induction. (**B**) Heatmaps visualizing H3K27Me3 decoration at *de novo* enhancers at different hours pi. of C10 cells. Window size: 6000 bp, bin size: 100. Right panel: coverage of H3K27Me3 around *de novo* enhancers. (**C**) Screenshots of selected enhancers, showing C/EBPα, PU.1, P300, H3K4Me1, H3K27Ac and H3K27Me3 profiles in C10 cells. (**D+E**) Median mRNA levels of genes nearest *pre-existing* or *de novo* enhancers during transdifferentiation of C10 cells (D) and primary pre-B cells (E). Statistical analysis by Wilcoxon test; one asterix, $p<0.01$, two asterixes $p<0.001$.

Supplemental Figure S6. Enhancer activity of macrophage enhancers during hematopoiesis.

(A+B) Bar graph showing the percentage of indicated enhancer type (see text and Figure 2) intersecting with enhancers either decorated with H3K4Me1 or H3K27Ac in various hematopoietic progenitors and differentiated cells. **(C)** Distribution of mRNA values of genes associated with *pre-existing* or *de novo* type enhancers in various hematopoietic progenitors and differentiated cells. (**D**) Similar as panel C but using cell samples prepared in slightly different ways and analyzed for gene expression by Affymetrix arrays (Di Tullio et al. 2011).

Supplemental Movie S1.

Transdifferentiation in action. The video shows a culture of C10 cells labeled with green fluorescent protein (GFP) surrounded by red fluorescent yeast (*Candida albicans*). As the transcription factor C/EBPα is activated within the B cells these aggregate and turn into macrophages that ingest the yeast, so that 51 hours after activation all pathogens became eaten. The microscope used for the acquisition was a Zeiss Cell Observer HS.

Supplemental Tables

Table S1: Summary of peak calling statistics of transcription factors and chromatin marks by HOMER.

Table S2: Peaks characteristics, annotation and gene expression of nearest genes of C/EBPα enriched regions in induced macrophages (iMΦ).

Table S3: Genomic coordinates of USCS screenshot examples and tested loci for ChIP (Refseq mm9/10).

Table S4: Primer sequences

Supplemental Experimental Procedures

Antibodies for ChIP-seq

Antibodies against C/EBPα (sc-61), C/EBPβ (sc-150), PU.1 (sc-352) and P300 (sc-585) were purchased from Santa Cruz Biotechnologies and against EBF1 (abnova1879) from Abnova. An antibody against H3K4Me1 (037-050) was purchased from Diagenode; antibody to H3K27Ac (ab4729) was purchased from Abcam (Cambridge UK) and to H3K27Me3 (17-622) from Millipore.

MNase-seq

To generate mononucleosomal DNA fragments, C10 cells were washed and resuspended in Buffer A (15 mM Tris-HCl pH: 7.5, 60 mM KCl, 15 mM NaCl, 2 mM CaCl2, 150 mM Sucrose, 0.15 mM spermin and 0,5 mM spermidin, lysolecithin at 0,5 mg/ml) for 1 min at 37 ° C. Then 30, 90, 200 and 300 units/ml of MNase were added and incubated for 2 min at 37° C. After stopping the reaction with 40 mM EDTA cells were lysed in Buffer B (50 mM Tris-HCl, pH: 8, 20 mM EDTA, 1% SDS), treated with RNase A and Proteinase K, DNA extracted with phenol-chlorophorm and precipitated. The DNA was run on an agarose gel (1% in TAE 1x) and the mononucleosomal band was excised, purified and used to prepare libraries.

Processing of ChIPseq and MNase data

To detect enriched regions we used HOMER (http://homer.salk.edu/homer/ngs/index.html) (Heinz et al. 2010) with the following settings: For factor binding (C/EBPα, C/EBPβ, PU.1, P300 and EBF1)

the peak localization/shape option was set to center and the style option to factor. For H3K4Me1, H3K4Me3 and H3K27Ac chromatin marks the style option was set to histone, the size option was set to 1000 and the minDist option was set to 1000. For H3K27Me3, the style option was set to histone, the size option was set to 5000 and the minDist option was set to 5000.

An overlap between C/EBPα enriched regions was defined by requiring the start or end position of an enriched region to lie between the start and end position of another enriched region (i.e. a minimal overlap of 1 bp). Coordinates of the shared region were merged and defined by the lowest and highest boundary. In this way, non-redundant binding sites for C/EBPα were identified by combining enriched regions of indicated time points post induction. Intersection of these non-redundant C/EBPα regions with histone marks required an overlap of 1 bp. Because coordinates of enriched C/EBPα regions were identified with the center option (see HOMER: http://homer.salk.edu/homer/ngs/index.html) for details and Supplemental Table 1, Supplemental Table 2: length of enriched regions), which defines a minimal centered region of the peak, intersection of these regions with other enriched regions of selected factors was defined as being in the proximity of maximally 1000 bp relative to start or end coordinate.

Heatmaps and average binding profiles

The position of aligned reads was extended by adding 150 bp to 5-3' aligned reads or subtraction of 150 bp from the position of 3-5' aligned reads. Data files in bed format were converted to Wig format using 'in house' awk scripts and subsequently converted to BigWig files using the 'wigToBigWig' utility from USCS and visualized by the USCS browser.

To generate heatmaps of factor binding or chromatin marks selected regions were centered on C/EBPα binding 48 hours post induction (hours pi) and extended 3000 bp up- and down-stream. For each 6000 bp region the coverage of aligned tags was calculated at 1 bp resolution and summed per 100 bp bin relative to the central position using Bedtools (Quinlan and Hall 2010) and "in house" awk /perl scripts. Matrix files were visualized using Treeview (Saldanha 2004).

To generate average profiles of factor binding or chromatin modifications selected regions were centered on the median position of the non-redundant C/EBPα binding sites and extended 3000 bp up and downstream. The average coverage was calculated per 1 bp bin relative to the central position. The maximum coverage value within the extended region (6000 bp) of each profile was plotted in either column or line graphs. For H3K27Me3 the coverage value of the center position was plotted.

To generate average profiles of MNase treated chromatin selected regions were centered on PU.1 at 48 hours pi. For each MNase profile we calculated the median value within the 6000 bp window and applied a median background subtraction. Control MNase profiles were generated by selecting random sites within 6000 bp used to generate PU.1 centered average MNase profiles. Graphs were based on the entire 6000 bp region.

GEO data tracks

GEO data tracks GSM1223648 (C/EBPα in primary macrophages) (Zhang et al. 2013) and GSM499030 (EBF1 in preB cells (Treiber et al. 2011), were re-aligned (mm9) using Bowtie without mismatches.

Series GSE60103 (H3K4Me1 and H3K27Ac in blood cells) (Lara-Astiaso et al. 2014), GSM1054815 (C/EBPα in Lsk) and GSM1187163 C/EBPα in GMP) (Hasemann et al. 2014) were re-aligned (mm10) using Bowtie2 standard settings. HOMER was used to detect enriched regions with the peak localization/shape option set to center and the style option set to factor for PU.1 peaks. For H3K4Me1 and H3K27Ac chromatin marks the style option was set to histone, the size option was set to 1000 and the minDist option was set to 1000. To convert mm9 to mm10 coordinates liftover utility of USCS was used with standard settings. GEO data GSE52373 (C/EBPα in primary B cells) (Di Stefano et al. 2013) and GEO data track mentioned above were converted to bigwig files using SRA toolkit from USCS and uploaded to the USCS browser.

Gene expression array analyses

To correlate factor binding with gene expression we used the gene expression databases described previously (Bussmann et al. 2009; Di Tullio et al. 2011; Lara-Astiaso et al. 2014). Each factor binding sites was associated with the mRNA level of the nearest gene defined in RefSeq (mm9). For subsets of sites, duplicate genes within each subset were removed and the median mRNA level of unique genes for each subset calculated. To correlate the spatial association of sustained C/EBPα binding sites with up-, down-regulated genes and all genes, genes were counted that are in the neighborhood of C/EBPα binding sites with windows of increasing size centered on these sites and expressed as a proportion of all genes for each group. Up-regulated genes were defined if the ratio of mRNA levels of 48 and 0 hours pi was ≥ 2, whereas down-regulated genes were defined as ≤ 0.5. To test for statistical differences in mRNA levels between subsets of genes we applied the Wilcoxon test, two-tailed, alpha level: 0.05.

Supplemental References

Quinlan AR, Hall IM. 2010. BEDTools: a flexible suite of utilities for comparing genomic features. *Bioinformatics* **26**: 841-842.

Saldanha AJ. 2004. Java Treeview--extensible visualization of microarray data. *Bioinformatics* **20**: 3246-3248.
