# Supplementary figures and images for: C/EBPα Activates Pre-existing and De Novo Macrophage Enhancers during Induced Pre-B Cell Transdifferentiation and Myelopoiesis
Source: Stem Cell Reports. 2015 Jul 30;5(2):232–47. doi: 10.1016/j.stemcr.2015.06.007 (PMC4618662; doi:10.1016/j.stemcr.2015.06.007)

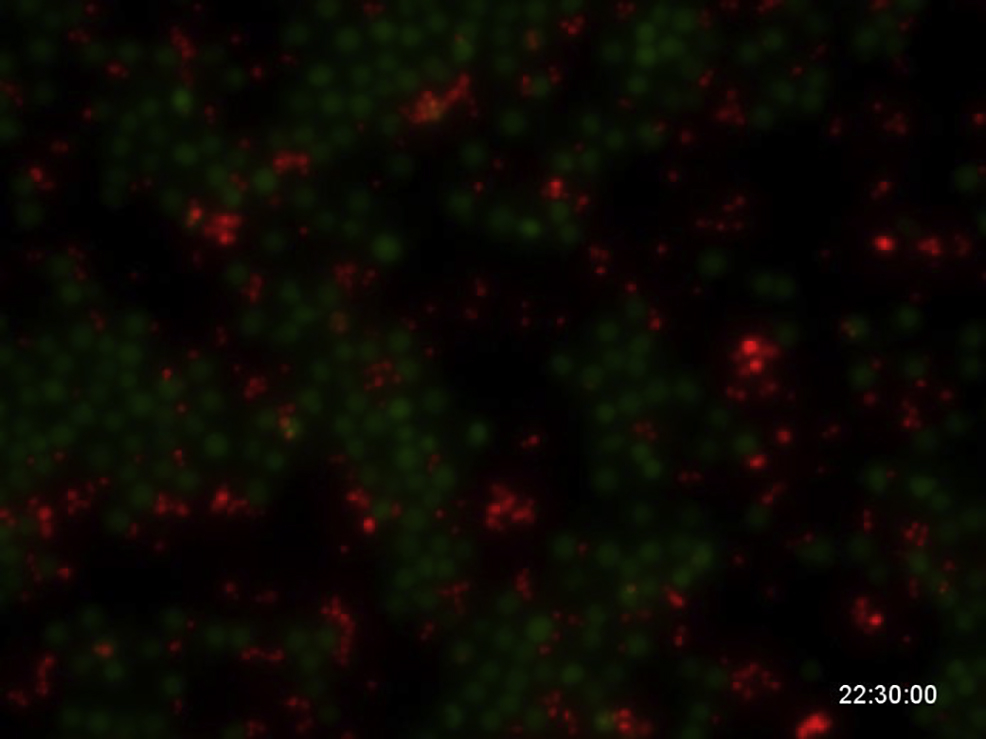

Supplement: Movie S1. Transdifferentiation in Action — The video shows a culture of C10 cells labeled with GFP surrounded by red fluorescent yeast (Candida albicans). As the TF C/EBPα is activated within the B cells, these aggregate and turn into macrophages that ingest the yeast, so that 51 hr after activation all pathogens were eaten. The microscope used for the acquisition was a Zeiss Cell Observer HS. [file mmc6.jpg]
